# Genetic structure of penicillin non-susceptible invasive Streptococcus pneumoniae in Colombia

**DOI:** 10.1099/mgen.0.001725

**Published:** 2026-05-19

**Authors:** Jamie Moreno, Johan F. Bernal, Monica Abrudan, Zonia Katerin Alarcon, Victor A. Medina, Anthony Underwood, Olga Sanabria, María Fernanda Valencia, Adriana Bautista, Alejandra Arévalo, Silvia Argimon, David Aanensen, Pilar Donado-Godoy, Carolina Duarte

**Affiliations:** 1 Grupo de Microbiología, Instituto Nacional de Salud, Bogotá, Colombia; 2 Global Health Research Unit for the Genomic Surveillance of Antimicrobial Resistance-Colombia, CI Tibaitatá, Corporación Colombiana de Investigación Agropecuaria (AGROSAVIA), Mosquera, Colombia; 3 Centre for Genomic Pathogen Surveillance, Big Data Institute, University of Oxford, Oxford, UK

**Keywords:** antimicrobial resistance (AMR), Colombia, clone, GPSC, MLST, serotype, *Streptococcus pneumoniae*

## Abstract

**Background.** Infections caused by *Streptococcus pneumoniae* are a public health problem worldwide, and penicillin-non-susceptible isolates are a priority for the World Health Organization, which requires further research and development of new antibiotics and vaccines.

**Aim.** To describe the global pneumococcal sequence clusters (GPSCs) among penicillin non-susceptible *S. pneumoniae* isolates obtained from patients with invasive pneumococcal disease in Colombia after the introduction of PCV10 (Synflorix, GlaxoSmithKline) to generate data on the genetic structure of pneumococcal populations with different antimicrobial susceptibilities.

**Methods.** A subset of 313 pneumococcal isolates with values of Minimum Inhibitory Concentration to penicillin of ≥0.125 µg ml^−1^, collected through the National Reference Laboratory at the National Health Institute of Colombia, were characterized by whole-genome sequencing to determine the GPSCs, serotypes, sequence types, antimicrobial resistance determinants and pilus islets.

**Results.** Most of the isolates (80%, *n*=251) were clustered in seven GPSCs: multidrug-resistant GPSC1 (40.6%, *n*=127) conformed principally to serotype 19A isolates and ST320, GPSC5 (10.5%, *n*=33) associated with ST338 and isolates of serogroups 23 and 6, GPSC13 (7.3%, *n*=23) which clustered 68% of 6A isolates, GPSC10 (7.0%, *n*=22) with 19A and 24F isolates, GPSC6 (5.4%, *n*=17) related to ST156, GPSC9 (4.8%, *n*=15) conformed to isolates 15A, 19 F/A and 6A and GPSC47 (4.5%, *n*=14) with 6C isolates. Only three isolates with PCV10 serotypes were recovered from children younger than 5 years old.

**Conclusion.** The results revealed changes in the population structure expected after PCV10 introduction, with GPSC1 being the most important clone due to its association with multidrug resistance. Genome sequencing of clinical multidrug-resistant isolates contributes to elucidating the antibiotic resistance mechanism and understanding the global pneumococcal population structure.

**Impact Statement**

The emergence of penicillin non-susceptible isolates has been attributed to the effects of antibiotic selective pressure, the clonal expansion of multidrug-resistant lineages and serotype replacement following the introduction of pneumococcal conjugate vaccines. This study provides the first comprehensive genomic characterization of penicillin non-susceptible pneumococci in Colombia, through the generation and analyis of the whole-genome sequences from 313 invasive pneumococcal isolates collected through the national surveillance system. We show that a small number of global pneumococcal sequence clusters (GPSCs), especially the multidrug-resistant GPSC1 and GPSC10 lineages dominated by serotype 19A, drive the burden of resistance. Our findings reveal specific patterns of antimicrobial resistance lineage, serotype replacement and clonal expansion following the introduction of PCV10, providing critical baseline data before the transition to PCV13. These results highlight the importance of sustained genomic surveillance to detect emerging non-vaccine multidrug-resistant lineages – such as GPSC5, GPSC9 and GPSC47 – that may challenge future vaccine impact. This study enhances the global comprehension of pneumococcal population dynamics and offers actionable evidence for public health decision-making within the region.

## Data availability

Raw sequence data were deposited at the European Nucleotide Archive under the bioproject accession number PRJEB76797. Specific accession numbers are listed in the supplementary file.

## Introduction

*Streptococcus pneumoniae* is a common cause of pneumonia, meningitis and other severe infections in children and elderly adults and is estimated to be responsible for 9 million cases of disease and more than 829,000 deaths per year among children under 4 years of age, with significant disease burden in low- and middle-income countries [1,2]. The polysaccharide capsule of *S. pneumoniae* is structurally diverse and a major virulence factor, which has enabled the identification of more than 100 serotypes [3]. The prevalence of different serotypes can be altered when new selective pressure is introduced, such as pneumococcal vaccines or by the antibiotic selection of resistant bacteria [3]. *S. pneumoniae* is among the high-priority pathogens that the World Health Organization monitors for antimicrobial resistance (AMR) through the Global Antimicrobial Resistance and Use Surveillance System [4], and penicillin-non-susceptible strains are known as increasingly drug-resistant pathogens that require further research and development of new antibiotics [5].

Pneumococcal conjugate vaccines (PCVs) have demonstrated efficacy against vaccine-preventable diseases, including serotypes responsible for invasive pneumococcal disease. However, post-vaccine introduction, there has been an increase in non-vaccine serotypes replacing the serotypes covered by the vaccine, and in some cases, vaccine serotypes escape the vaccine-induced immunity by capsule switching [6,7]. The Global Pneumococcal Sequencing project (http://www.pneumogen.net/gps/) defined 990 pneumococcal lineages (https://www.pneumogen.net/gps/GPSC-ST.html), named the global pneumococcal sequence clusters (GPSCs) to study pneumococcal serotypes, antibiotic resistance and invasiveness, in the context of genetic background [8]. Sequencing data can be used to model serotype replacement, predict the impact of a vaccine, design novel vaccines and identify emerging lineages with enhanced virulence or antimicrobial resistance [8,9].

In Colombia in 1994, the SIREVA II network surveillance system for the causative agents of pneumonia and meningitis [10] was established to identify serotypes, antibiotic resistance and circulation of international clones among isolates recovered from patients with invasive pneumococcal disease. The data obtained have served as evidence for decision-making in public health policy. In 2009, PCV7 was introduced free of charge in the National Immunization Program of Colombia, substituted by PCV10 (Synflorix, GlaxoSmithKline) in January 2012 and recently replaced by PCV13 for populations born on or after 1 May 2022, on a 2+1 schedule at 2, 4 and 12 months of age. As a result of the introduction of PCV7/10 vaccines, the post-vaccination annual national surveillance data exhibited a reduction in PCV10 serotypes, whereas an increase in serotypes 19A, 3 and non-PCV13 was observed [11].

The implementation of the Global Health Research Unit on Antimicrobial Resistance (GHRU-AMR) at the Corporación Colombiana de Investigación Agropecuaria (AGROSAVIA), funded by the National Institute of Health Research and supported by the Center of Genomic Pathogen Surveillance at Oxford University, UK [12], enabled the generation of genomic data complemented by phenotypic surveillance, providing more detailed insights into the epidemiology of resistant *S. pneumoniae* isolates in Colombia. Additionally, the Ministry of Health guidelines recommend the administration of a beta-lactam antibiotic (amoxicillin) for the treatment of community-acquired pneumonia [13]. Therefore, this study aimed to describe the GPSCs circulating among penicillin non-susceptible *S. pneumoniae* isolates obtained from patients with invasive pneumococcal disease in Colombia after PCV10 introduction and before the introduction of PCV13. The data generated will provide a baseline for delineating the genetic structure of the pneumococcal population that is not susceptible to penicillin, assessing vaccine impact and inferring relationships between Colombian isolates.

## Methods

### Bacterial isolates

From 2015 to 2018, the National Reference Laboratory at the National Health Institute of Colombia received invasive *S. pneumoniae* isolates (*n*=2,069) from 23 political administrative divisions of Colombia (departments and district capital cities) as part of the SIREVA II [10]. The isolates were confirmed by colony morphology, optochin susceptibility and solubility in bile. Serotyping was performed following the Neufeld Quellung reaction scheme. The antimicrobial susceptibility of the isolates was determined using the broth microdilution method according to the Clinical and Laboratory Standards Institute (2022). Approximately half of the isolates (*n*=1,002; 48.4%) exhibited values of minimum inhibitory concentration (MIC) to penicillin of ≥0.125 µg ml^−1^, which is the reference value for non-susceptible to penicillin meningitis isolates. A set of 313 (31.2%) isolates was selected by convenience sampling for sequencing, considering isolates with a penicillin MIC of ≥0.125 µg ml^−1^, the frequencies of the serotypes in each year of the study period and the year of collection. Multidrug resistance (MDR) was defined as resistance to three or more classes of antibiotics.

### Whole-genome sequencing

From bacteria in the exponential growth phase in modified Todd-Hewitt broth culture, the DNA of bacteria was extracted by the QIAamp DNA mini-kit (QIAGEN, Germany), using the protocol for isolation of genomic DNA from bacterial cultures and following the manufacturer’s instructions. The quantity and quality of DNA were measured using a NanoDrop^™^ 2000 spectrophotometer (Thermo Scientific, USA) and Qubit 4.0 fluorometer (Invitrogen, USA); the integrity was evaluated on agarose gel electrophoresis (0.8% w/v). The sequencing process was performed using the Illumina HiSeq-X10 platform (San Diego, CA, USA) at the Wellcome Trust Sanger Institute (Hinxton, UK). All raw reads were processed for quality control, and the parameters are available in the open-access GHRU-AMR versioned protocols (https://www.protocols.io/view/ghru-genomic-surveillance-of-antimicrobial-resista-bp2l6b11kgqe/v4). The major quality control parameters of the sequences are available in Table S1 (available in the online Supplementary Material).

### Data analysis

The raw reads that complied with the quality control criteria were processed using GHRU-AMR versioned protocols for the antimicrobial resistance determinants and multilocus sequence type (MLST). Other secondary processes, such as *in silico* serotyping, GPSC and penicillin-binding protein (PBP) profiles, were defined using SeroBA (v1.0.2), popPUNK (v2.6.1) and PBP reference (https://github.com/BenJamesMetcalf/Spn_Scripts_Reference) software, respectively, as those implemented on the PathogenWatch platform [14]. Virulence factors were assessed using ARIBA (v2.14.4) software with the entire Virulence Factor Data Base (VFDB). SNPs were called based on the mapping to the reference genome of the *S. pneumoniae* strain (ATCC 700669 NC_011900.1) [15], and a maximum-likelihood phylogeny was inferred using IQ-TREE (v1.6.8) software with the GTR+G model and 1,000 bootstrap replicates, as described in the GHRU-AMR protocol (dx.doi.org/10.17504/protocols.io.bp2l6b11kgqe/v4). High-quality views of the trees and data were rendered using the ggtree2 R package (https://bioconductor.org/packages/release/bioc/html/ggtree.html), and the Microreact project is available at https://microreact.org/project/rYpCeEHXgMqfX44raUkuia-penicillin-non-susceptible-streptoccocus-pneumoniae-from-national-health-institute-of-colombia-2015-2018.

## Results

Among the *S. pneumoniae* penicillin non-susceptible isolates (*n*=313) recovered from 23 departments (territorial entities), 70.0% (*n*=129) of the isolates were from six departments (Bogotá, Valle, Antioquia, Risaralda, Bolívar and Boyacá). Among all the isolates, 56.2% (*n*=176) were from males, and 43.5% (*n*=136) were from females; most were from children <2 years old (17.9%, *n*=26) and adults >60 years old(28.4%, *n*=89). The most frequent sources of bacterial isolation were blood (70.3%, *n*=220) and cerebrospinal fluid (19.5%, *n*=61), and the most reported clinical diagnoses were pneumonia (30.3%, *n*=95), meningitis (25.9%, *n*=81) and sepsis (20.4%, *n*=64). Similarly, pneumonia (48.4%, *n*=46) was the most frequent diagnosis in children younger than 5 years, and meningitis (43.2%, *n*=35) was most frequently diagnosed in adults older than 50 years (Table 1).

**Table 1.** Distribution of non‑penicillin‑susceptible invasive *S. pneumoniae* isolates by age group, clinical diagnosis, source of isolation and GPSC, in patients with invasive disease in Colombia, 2015–2018

Age group (years)	Clinical diagnosis (*n*, %)					Isolate source (*n*, %)				GPSC (*n*)							
	Pneumonia	Meningitis	Sepsis	Other	No data	Blood culture	CFS*	Pleural fluid	Others	1	5	13	10	6	9	47	Others
< 2	26 (27.4)	11 (13.6)	10 (15.6)	4 (9.3)	5 (16.7)	37 (16.8)	10 (16.4)	9 (28.1)	1	30	3	2	8	1	–	1	11
2 to <5	20 (21.0)	3 (3.7)	9 (14.1)	10 (23.3)	9 (30.0)	38 (17.3)	2 (3.3)	11 (34.4)	1	34	2	1	4	2	2	–	6
5 to <14	6 (6.3)	8 (9.9)	3 (4.7)	7 (16.3)	2 (6.7)	17 (7.7)	8 (13.1)	1 (3.1)	1	6	3	4	1	4	1	2	5
14 to <50	10 (10.5)	20 (24.7)	10 (15.6)	4 (9.3)	2 (6.7)	33 (15.0)	15 (24.6)	2 (6.2)	1	19	9	3	1	2	1	2	13
50 to <60	7 (7.3)	10 (12.3)	9 (14.1)	4 (9.3)	4 (13.3)	25 (11.4)	7 (11.5)	2 (6.2)	1	12	4	2	1		3	1	11
>60	24 (25.3)	25 (30.9)	23 (35.9)	13 (30.2)	4 (13.3)	67 (30.4)	16 (26.3)	6 (18.7)	4	26	10	6	7	8	8	8	16
No data	2 (2.1)	4 (4.9)	0	1 (2.3)	0	3 (1.4)	3 (4.9)	1 (3.1)	1	–	2	5	–	–	–	–	–
Total	95 (30.3)	81 (25.9)	64 (20.4)	43 (13.7)	30 (9.7)	220 (70.3)	61 (19.5)	32 (10.2)	10	127	33	23	22	17	15	14	62

CSF, cerebrospinal fluid.

**Fig. 1.**
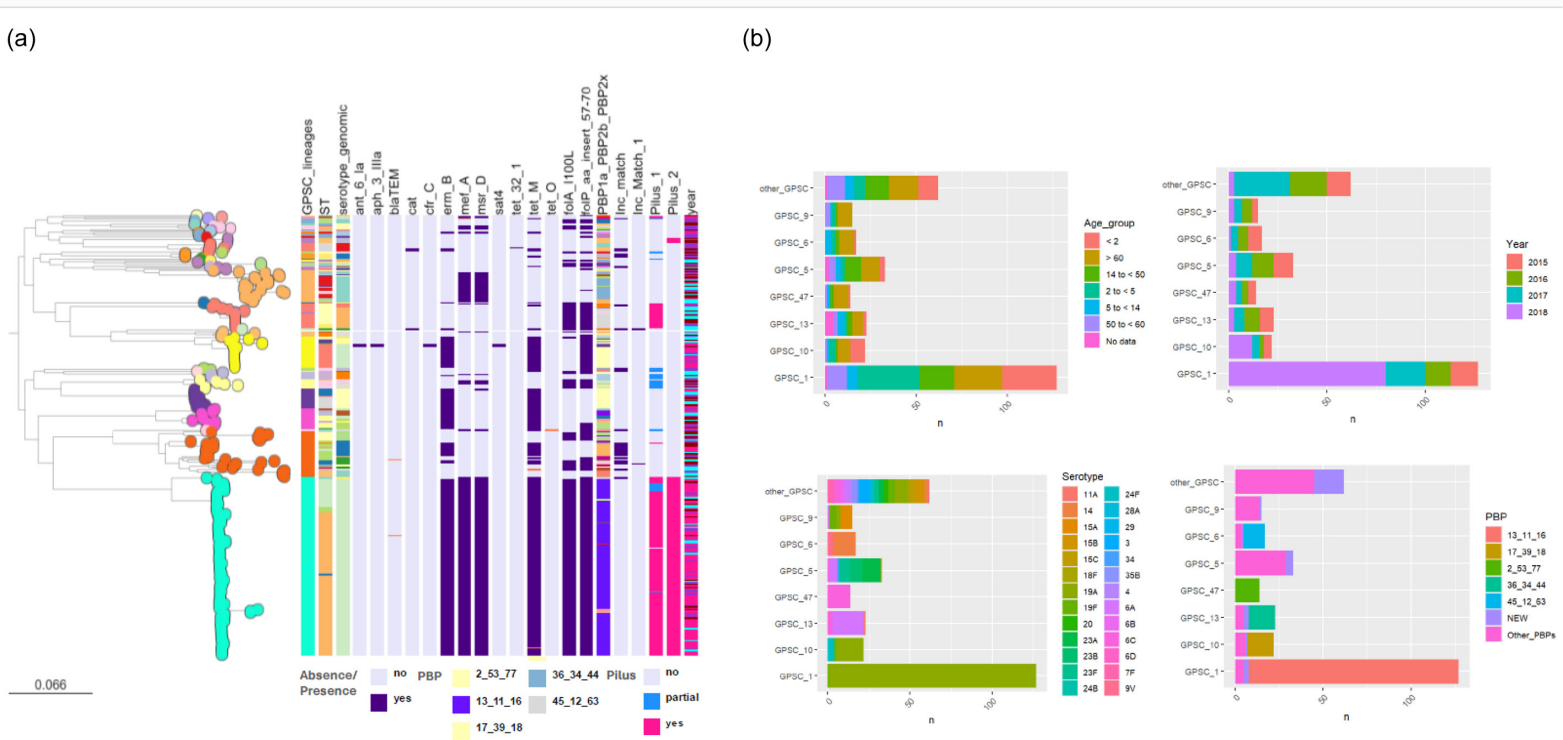
Phylogeny of GPSCs of non-penicillin-susceptible *S. pneumoniae* isolates recovered from patients with invasive disease in Colombia in the period from 2015 to 2018. Leaf nodes on the tree are colour-coded according to GPSC. Microreact: https://microreact.org/project/rYpCeEHXgMqfX44raUkuia-penicillin-non-susceptible-streptoccocus-pneumoniae-from-national-health-institute-of-colombia-2015-2018. Legend: The figure displays GPSCs, STs, serotypes, PBP profiles, sample origins and the presence of selected resistance determinants (*ermB*, *mefA*, *msrD*, *tetM*, *folA* and *folP* genes).

Among the total isolates (*n*=313), 33 GPSCs were identified, and 5 isolates were not assigned (Fig. 1). Eighty per cent of the isolates were clustered in seven GPSCs: GPSC1 (40.6%, *n*=127), GPSC5 (10.5%, *n*=33), GPSC13 (7.3%, *n*=23), GPSC10 (7.0%, *n*=22), GPSC6 (5.4%, *n*=17), GPSC9 (4.8%, *n*=15) and GPSC47 (4.5%, *n*=14). Sixty-four known and 22 new sequence types (STs) were identified, of which ST320 (31.9%, *n*=100), ST1451 (6.4%, *n*=20) and ST276 (5.4%, *n*=17) were the most frequent. Twenty-six serotypes were recognized, and the most prevalent were 19A (49.2%, *n*=134), 6A (8.9%, *n*=28) and 6C (6.7%, *n*=21) (Table 2).

**Table 2.** Characteristics of predominant GPSCs among non-penicillin-susceptible invasive isolates of *S. pneumoniae* recovered from patients with invasive disease in Colombia in the period from 2015 to 2018

Molecular typing						Phenotype resistance from MIC (μg ml^−1^)						Genotype resistance^†^								
GPCS	n (%)	Serotype	*n* (%)	ST	n (%)	PEN MIC		ERY MIC ≥0.5	SXT MIC ≥2	TET MIC ≥2	CHL MIC ≥8	PBP profile		*ermB*	*mefA*	*msrD*	*tetM*	*cat*	*folP*	*folA/P*
							n (%)													
1	127 (40.6)	19A	126 (40.3)	320	100 (31.9)	≤2	5 (1.6)	127 (54.7)	126 (65.3)	123 (60.6)	2 (25.0)	Others (*n*=4)	8 (2.6)	119 (64.0)	127 (78.9)	127 (78.9)	126 (63.3)	0	1 (2.5)	126 (69.6)
		19F	1 (0.3)	1,451	20 (6.4)	≥4	122 (38.9)					13-11-16	119 (38.0)							
				Others (*n*=3)	7 (2.2)															
5	33 (10.5%)	23A/B/F	11 (3.5), 7 (2.2), 7 (2.2)	338	10 (3.2)	≤2	33 (10.5)	12 (5.2)	14 (7.2)	14 (6.9)	0	19-1-24	9 (2.9)	12 (6.4)	0	0	13 (6.5)	0	3 (7.5)	13 (7.2)
		6(A/C	5 (1.6), 1 (0.3)	2,372	6 (1.9)	≥4	0					0-1-1	6 (1.9)							
		15C/34	1 (0.3), 1 (0.3)	Others (*n*=11)	17 (5.4)							Others (*n*=9)	18 (5.7)							
13	23 (7.3%)	6 A/C/B	19 (6.1), 2 (0.6), 1 (0.3)	473	11 (3.5)	≤2	23 (7.4)	21 (9.1)	0	0	0	36-34-44	15 (4.8)	0	21 (13.0)	21 (13.0)	0	0	1 (2.5)	0
		11A	1 (0.3)	1,717	2 (0.6)	≥4	0					2-34-44	2 (0.6)							
				Others (*n*=8)	10 (3.2)							Others (*n*=4)	6 (1.9)							
10	22 (7.0%)	19 A/F	17 (5.4), 1 (0.3)	276	17 (5.4)	≤2	8 (2.6)	22 (9.5)	10 (5.2)	21 (10.3)	1 (12.5)	Others (*n*=5)	8 (2.6)	20 (10.7)	0	0	21 (10.5)	0	21 (52.5)	1 (0.6)
		24F/B	3 (1.0), 1 (0.3)	230	2 (0.6)	≥4	14 (4.5)					17-39-18	15 (4.8)							
				Others (*n*=3)	3 (1.0)															
6	18 (5.7%)	14	15 (4.7)	156	14 (4.7)	≤2	6 (1.7)	1 (0.4)	17 (8.8)	1 (0.5)	0	45-12-63	12 (3.8)	0	1 (0.6)	1 (0.6)	1 (0.5)	0	0	17 (9.4)
		9V	3 (1.0)	18,093	3 (1.0)	≥4	12 (3.8)					15-12-18	3 (0.9)							
												Others (*n*=3)	2 (0.6)							
9	15 (4.8%)	15A	7 (2.2)	63	7 (2.2)	≤2	14 (4.5)	15 (6.5)	5 (2.6)	15 (7.4)	2 (25.0)	24-27-179	5 (1.6)	15 (8.1)	0	0	15 (7.5)	0	5 (12.5)	0
		19 F/A	4 (1.3), 3 (1.0)	861	4 (1.3)	≥4	1 (0.3)					24_27_192	4 (1.3)							
		6A	1 (0.3)	Others (*n*=4)	4 (1.3)							Others (*n*=4)	6 (1.9)							
47	14 (4.5%)	6C	14 (4.5)	386	8 (2.6)	≤2	14 (4.5)	14 (6.0)	0	11 (5.4)	0	2_53_77	14 (4.4)	14 (7.5)	0	1 (0.6)^‡^	11 (5.2)	0	0	0
				4,310	5 (1.6)	≥4	0													
				11,793	1 (0.3)															
Others (*n*=27)	62 (19.8%)			34	62 (19.6)	≤2	57 (18.2)	32 (13.8)	21 (10.9)	18 (8.9)	3 (37.5)	*n*=37	62 (19.7)	6 (6.3)	12 (7.5)^‡^	11 (6.8)^‡^	12 (6.1)	3 (100)	9 (22.5)	24 (13.2)
						≥4	5 (1.6)													
Total	313						313	232	193	203	8		313	186	161	161	199	3	40	181

*Penicillin resistance was defined as an MIC of ≥0.12 μg ml−1, according to meningitis breakpoints.

†Pneumococcal isolates that exhibited intermediate and full resistance to STX and TET were categorized as resistant isolates.

‡Partial gene deletion.

CHL, chloramphenicol; ERY, erythromycin; PEN, penicillin; SXT, sulfamethoxazole-/trimethoprim; TET, tetracycline.

Forty-six per cent (*n*=127) of the isolates belonged to GPSC1, of which 50.4% (*n*=64) were recovered from children under 5 years old. Apart from a serotype 19F isolate (ST236), all the GPSC1 isolates (99.2%, *n*=126) were serotype 19A with ST320 (78.7%, *n*=100), ST1451 (15.7%, *n*=20) and novel ST13455 (3.1%, *n*=4), as the most frequent sequence types. Isolates with ST1451 and ST13455 formed a phylogenetic sub-cluster. All the isolates were phenotypically MDR (Table 2); PBP profile 13-11-16 was identified in 119 (93.7%) isolates with penicillin MICs ranging from 4.0 to 8.0 µg ml^−1^. All the isolates carried *ermB*, *mefA*, *msrD* and *tetM* genes and alterations in the *folA* and *folP* genes (a non-synonymous mutation in the *folA* gene resulting in an I100L substitution and an insertion at position 199, or mutations at positions 178–180 in the *folP* gene), except for the 19F isolate, which carried *mefA*, *msrD* and *tetM*.

GPSC-5 was mainly recovered from patients older than 50 years (*n*=14) and consisted of 33 isolates principally associated with ST338 (serotype 23 A, *n*=8; serotype 23F, *n*=2), ST2372 (serotype 23B, *n*=6), ST15260 (serotype 6A, *n*=5), and ST15243 (serotype 23F, *n*=3). Twenty-seven per cent (*n*=9) of the isolates had novel STs. The MICs of penicillin for the GPSC5 isolates ranged from MICs of 0.25 to 1.0 µg ml^−1^, and the main PBPs were 19-1-24 (*n*=9), 0-1-1 (*n*=6) and 36-1-44 (*n*=5). The resistance determinants, *ermB* and *tetM* genes were detected in 12 isolates and were associated mainly with serotype 23A (*n*=10).

GPSC13 was confirmed in 23 (7.3%) isolates, 19 (82.6%) of which belonged to serotype 6A, which represented 68% of the isolates of this serotype. The most frequent ST was ST473 (47.8%, *n*=11) and its locus variants ST471 (*n*=1) and ST1717 (*n*=2). Additionally, four novel STs were identified: ST15244, ST15258, ST15263 and ST15266. ST15244 was also identified in an isolate serotype 11A. The remaining isolates were 6B (ST15442, new ST) and 6C (ST1135 and ST5679). The MICs of penicillin were 0.125 to 1.0 µg ml^−1^; most of the serotype 6A isolates had a PBP 36-34-44 profile (*n*=15, 78.9%), and 21 (91.3%) harboured *mefA-msrD* macrolide resistance determinants.

GPSC10 grouped 22 (7%) isolates were recovered mainly from children under 2 years of age (*n*=8) and older adults (>60, *n*=7). Serotypes 19A (ST276, *n*=17) and 24F with three isolates (ST230, ST4256 and ST4677) were most frequent. All serotype 19A isolates belonging to GPSC10 (77.3%, *n*=17) were MDR and had PBP 17-39-18 with a phenotypic penicillin MIC of 2.0 to 8.0 µg ml^−1^; 20 (90.1%) GPSC10 isolates harboured the *ermB* and *tetM* genes.

GPSC6 contained 17 isolates of serotypes 14 (*n*=14) and 9V (*n*=3), which were recovered mainly from patients older than 60 years (*n*=8). ST156 or a single locus variant of ST156 was observed in 14 (82.4%) and three (17.6%) isolates, respectively. Twelve ST156 isolates had phenotypic penicillin MICs of 2.0 to 4.0 µg ml^−1^ associated with a PBP 45-12-63 profile, all the GPSC6 isolates exhibited trimethoprim/sulfamethoxazole (SXT) resistance due to mutations within the *folP/A* genes, and one isolate harboured the *tetM* and *ermB* genes.

GPSC9 comprised 15 isolates (4.8%) belonging to serotypes 15A (*n*=7), 19F (*n*=4), 19A (*n*=3) and 6A (*n*=1), which exhibited penicillin MICs ranging from 0.125 to 1.0 µg ml^−1^. ST63 [46.6%; 15A (*n*=7) and 19A (*n*=2) serotypes] and ST861 [26.7%; 19F (*n*=3) and 6A (*n*=1) serotypes] were most frequent, and three new STs (ST15246, ST15265 and ST18097) were identified in this study. PBPs 24-27-179 and 24-27-142 were present in isolates 15A (*n*=5) and 19F (*n*=3), respectively. All isolates were resistant to erythromycin and tetracycline. The *ermB* and *tetM* genes were present in all the isolates, and *folP* was present in five 15A isolates with SXT resistance.

GPSC47 was confirmed in serotype 6C isolates with ST386 (*n*=8), ST4310 (*n*=5) and ST11793. All the isolates had PBP 2-53-77 (MICs of 0.125 to 0.5 µg ml^−1^), *ermB* and *tetM*; however, in two isolates, the *tetM* gene had a premature stop codon at position 634 or 636 of the reference gene NG_048230.1, and the isolates were phenotypically susceptible to tetracycline.

Other GPSCs containing fewer than ten isolates were also identified (Table S2). GPSC48 was confirmed for isolates ST3557 with 15B/C (*n*=6) and 19A (*n*=1) serotypes. All the isolates with serotype 3 (*n*=6) were ST180 and clustered in GPSC12, and two had resistance to chloramphenicol (*cat*) and tetracycline (*tetM*).

Forty-six isolates had serotypes included in PCV10, of which 28 were grouped in GPSCs that contained only PCV10 serotypes, mainly GPSCs 6, 15 and 2. Fifteen isolates were assigned to GPSCs comprising both PCV10 and non-PCV10 serotypes; in three cases, GPSCs could not be determined.

## Discussion

Antimicrobial resistance in *S. pneumoniae* changes over time and across countries, depending on factors such as PCV implementation and coverage, serotype distribution, local antibiotic consumption and the circulation of distinct clonal lineages, among others [8,16]. The identification and prevalence of GPSCs associated with drug resistance could help identify genomic lineages responsible for resistance maintenance. The results of the WGS of Colombian pneumococcal isolates with values of MIC to penicillin of ≥0.125 µg ml^−1^ revealed that the MDR pneumococcal lineage GPSC1 was primarily responsible for antimicrobial resistance, followed by the dispersion of GPSC5, GPSC13 and GPSC10.

Multidrug-resistant GPSC1 was the predominant cluster in this study, comprising isolates that were almost exclusively serotype 19A, most of which belonged to sequence type ST320. ST320 is a double-locus variant (DLV) of the Taiwan19F-14 (ST236) clone, primarily associated with the vaccine serotype 19F [17]. ST320 has increasingly been identified as a cause of invasive pneumococcal disease during the post-PCV7/10 period in several countries worldwide, including Colombia [6,18, 19]. The presence of the ST1451 subclade, a single-locus variant (SLV) of ST230 associated with drug resistance and first reported in isolates recovered in the USA in 2000 [17], provides evidence of its international spread and persistence over time. ST1451 has been detected in non-invasive isolates in Finland [20], in a case of vaccine failure [21], in one invasive isolate in Argentina [22] and now in Colombia. The novel ST13455, a DLV of ST320, has so far only been identified in isolates from Colombia; however, its multidrug-resistant characteristics may facilitate its proliferation and establishment. The identification of the *ermB*, *mefA*, *msrD* and *tetM* genes in GPSC1 suggests the presence of Tn*2010*, a composite transposon of the Tn*916* family, which was previously identified in Colombian isolates with serotype 19A related to ST320 [23], and provides a competitive advantage for the growth and expansion of GPSC1.

GPSC5 is a heterogeneous lineage characterized by multiple MLSTs and includes both vaccine-associated and non-vaccine-associated serotypes [8]. The frequency of serotypes comprising GPSC5 has been shown to vary between countries. In this study, GPSC5 was associated with serotype 23A isolates, in contrast to Nigeria [24], where serotype 6A predominated, and England, where serotype 23B was the most frequent [25]. ST338 was characterized in serotype 23F isolates with low-level resistance to penicillin and susceptibility to other antimicrobial agents and was related to the ST of the PMEN clone Colombia23F-26 [26,27]. According to our results, this ST has been reported previously in isolates with serotype 23A, probably originated before the introduction of the PCVs by capsular switching, and was seen mostly among adults, possibly due to the cross-protectivity provided by the PCVs [28,29]. Additionally, our isolates carried the *ermB* and *tetM* genes; therefore, the expansion of this subclade could be favoured by its increased resistance to multiple antibiotics. Penicillin and SXT-non-susceptible serotype 23B GPSC5 isolates had ST2372 associated with a novel 23B1 genotypic variant identified as dominant in UK isolates [30]. In comparison, several countries reported serotype 23B isolates associated with ST172, a SLV of ST338 [27]. ST15260, a novel ST identified in this study, was determined in serotype 6A isolates, which is also a double variant locus of ST172 and associated with GPSC5 in 19A isolates from Argentina [22]. The lineage diversity of GPSC5 is high, as evidenced by the diversity of serotypes and the generation of novel STs with a common genetic background.

The study showed that several serotype 19A and all serotype 24F isolates belonged to GPSC10. GPSC10 is a multidrug-resistant pneumococcal lineage with both vaccine and non-vaccine serotypes and can mediate serotype replacement in the post-vaccine era with PCV7 and PCV10 [31,32]. Before PCV13, serotype 19A isolates of GPSC10 were a predominant cause of invasive disease in some European countries [31], but a rapid change in serotype composition within GPSC10 from 19A to 24F after PCV13 was observed in Spain, France, Israel and Argentina [22,31]. This suggests that following the introduction of PCV13 into the national immunization programme, changes in the composition of GPSC10 serotypes may occur, including a reduction in serotype 19A and an increase in resistant serotype 24F isolates. The impact of PCV13 on the 19A serotype and antibiotic-selective pressure could facilitate the adaptation and proliferation of serotype 24F isolates with an invasive disease potential similar to serotype 19A [33]. Therefore, continued molecular surveillance of pneumococcal isolates is necessary to monitor the introduction and spread of clones with antimicrobial resistance.

The lineage GPSC6 was identified in serotypes 14 and 9V isolates with ST156 and was recovered mainly from older patients. Serotype 14 had been identified as SXT and penicillin-resistant serotype related to the Spain9V-ST156 clone and was the leading cause of IPD in Colombia before the introduction of PCV7 [34]. During the post-PCV period, serotype 14 decreased in prevalence, especially in children younger than 2 years [11]. A retrospective study from 55 hospitals in Bogotá (Colombia) indicated that in adult patients hospitalized due to IPD, serotype 14 ranked third in frequency (5.8%) among all cases [35]. The persistence of GPSC6 in the adult population can be due to chronic medical conditions, particularly among elderly adults, which increase the risk of pneumococcal disease and incomplete establishment of herd protection in unvaccinated people.

According to the GPSC database (https://data.monocle.sanger.ac.uk/), GPSC13 is composed of serogroup six isolates, mainly the 6A serotype and ST473. ST473 was identified as the predicted founder of a clonal complex composed of serotype 6A and 6B isolates, which arose by recombinant replacement at the cps locus, resulting in a switch from serotype 6A to 6B [36]. This GPSC was observed as a minor lineage in 6A isolates from Argentina during the post-vaccine period [22].

In this study, GPSC9 was identified in vaccine and non-vaccine PCV13 serotypes. GPSC9 has shown an association between vaccine use and variation in capsular type expression, with a decrease in vaccine serotypes and an increase in non-vaccinal serotypes following the introduction of PCVs [36,39]. This lineage has been associated with MDR non-PCV 15 A/C serotypes, which have increased in several parts of the world following the implementation of PCV13 [37,38]. Additionally, GPSC9 can evade vaccines through loss of capsular expression, which is associated with the acquisition of the *psp*C (pneumococcal surface protein C) virulence factor within the cps locus [36].

GPSC47 was associated with an important MDR non-vaccinal type 6C. Because GPSC47 expresses PCV13 and non-PCV13 serotypes and MDR, it can adapt and continue to cause infections under selective pressure from vaccines and antibiotics [40,41]. Like those previously established in Brazil [42], serotype 3 (ST180) isolates were clustered in GPSC12. CC180 was related to an emerging clade II, which is now globally distributed, with a relatively high prevalence of antimicrobial resistance due to the presence of *erm*B, *tet*M and *cat* [43].

The limitations of this study were related to laboratory-based surveillance. These data may not reflect the national status of the genetic behaviour of the population of resistant *S. pneumoniae* in Colombia, and they might be affected by the underreporting of cases when notification of pneumococcal diseases is not mandatory. Additionally, the GPSC determination was based on isolates whose MIC of penicillin was ≥0.125 µg ml^−1^ and not on the total number of isolates, which provides an incomplete lineage structure. Another important limitation is that the collection of isolates includes only penicillin-resistant pneumococci and, therefore, does not include other penicillin-sensitive MDR patterns, such as SXT-CLO-TE. However, the results may reflect an overall trend in the lineages of *S. pneumoniae* in Colombia.

In conclusion, GPSC1 and GPSC10 significantly contributed to the dispersion of antibiotic-resistant serotype 19A isolates in our country during the post-PCV10 era. The recent introduction of PCV13 into Colombia’s vaccination schedules will likely reduce MDR serotype 19A in all GPSCs effectively. However, the selective pressure induced by PCV13 may lead to shifts in serotype composition through the expansion of pre-existing non-vaccine serotype variants within these or other lineages [44]. Non-vaccine MDR GPSC10 (24B/F), GPSC5 (23A/B, 15C, 34), GPSC9 (15A) and GPSC47 (6C) lineages should be considered a public health concern in Colombia since the post-PCV13 era in other countries has shown the importance of these emerging lineages on the trends of the invasive pneumococcal disease [43]. Understanding the lineages driving post-vaccine serotype expansion will help guide future pneumococcal disease prevention strategies. Therefore, it is essential to identify pneumococcal variants with the potential to evade vaccine-induced protection.

## Supplementary material

10.1099/mgen.0.001725 Uncited Table S1.

10.1099/mgen.0.001725 Uncited Table S2.

## Abbreviations

AMR: antimicrobial resistance
DLV: double-locus variant
GHRU-AMR: Global Health Research Unit on Antimicrobial Resistance
GPSCs: global pneumococcal sequence clusters
MDR: multidrug resistance
MIC: minimum inhibitory concentration
MLST: multilocus sequence type
PBP: penicillin-binding protein
PCVs: pneumococcal conjugate vaccines
SLV: single-locus variant
STs: sequence types
SXT: trimethoprim/sulfamethoxazole
