# Supplementary material for: Genetic structure of penicillin non-susceptible invasive Streptococcus pneumoniae in Colombia
Source: Microb Genom. 2026 May 19;12(5):001725. doi: 10.1099/mgen.0.001725 (PMC13186401; doi:10.1099/mgen.0.001725)
Supplement: Uncited Table S1. [file mgen-12-01725-s001.pdf]

List of *Streptococcus pneumoniae* isolates of the project PRJEB76797 (n=313)					
#	id	run_accession	sample_accession	tax_name	fastq_ftp
1	G20000379	ERR15948694	SAMEA120533183	1313	ftp.sra.ebi.ac.uk/vol1/fastq/ERR159/094/ERR15948694/ERR15948694_1.fastq.gz;ftp.sra.ebi.ac.uk/vol1/fastq/ERR159/094/ERR15948694/ERR15948694_2.fastq.gz
2	G20000377	ERR15948695	SAMEA120533182	1313	ftp.sra.ebi.ac.uk/vol1/fastq/ERR159/095/ERR15948695/ERR15948695_1.fastq.gz;ftp.sra.ebi.ac.uk/vol1/fastq/ERR159/095/ERR15948695/ERR15948695_2.fastq.gz
3	G20000368	ERR15948699	SAMEA120533178	1313	ftp.sra.ebi.ac.uk/vol1/fastq/ERR159/099/ERR15948699/ERR15948699_1.fastq.gz;ftp.sra.ebi.ac.uk/vol1/fastq/ERR159/099/ERR15948699/ERR15948699_2.fastq.gz
4	G20000362	ERR15948702	SAMEA120533175	1313	ftp.sra.ebi.ac.uk/vol1/fastq/ERR159/002/ERR15948702/ERR15948702_1.fastq.gz;ftp.sra.ebi.ac.uk/vol1/fastq/ERR159/002/ERR15948702/ERR15948702_2.fastq.gz
5	G20000354	ERR15948707	SAMEA120533170	1313	ftp.sra.ebi.ac.uk/vol1/fastq/ERR159/007/ERR15948707/ERR15948707_1.fastq.gz;ftp.sra.ebi.ac.uk/vol1/fastq/ERR159/007/ERR15948707/ERR15948707_2.fastq.gz
6	G20000337	ERR15948717	SAMEA120533160	1313	ftp.sra.ebi.ac.uk/vol1/fastq/ERR159/017/ERR15948717/ERR15948717_1.fastq.gz;ftp.sra.ebi.ac.uk/vol1/fastq/ERR159/017/ERR15948717/ERR15948717_2.fastq.gz
7	G20000302	ERR15948737	SAMEA120533140	1313	ftp.sra.ebi.ac.uk/vol1/fastq/ERR159/037/ERR15948737/ERR15948737_1.fastq.gz;ftp.sra.ebi.ac.uk/vol1/fastq/ERR159/037/ERR15948737/ERR15948737_2.fastq.gz
8	G20000293	ERR15948743	SAMEA120533134	1313	ftp.sra.ebi.ac.uk/vol1/fastq/ERR159/043/ERR15948743/ERR15948743_1.fastq.gz;ftp.sra.ebi.ac.uk/vol1/fastq/ERR159/043/ERR15948743/ERR15948743_2.fastq.gz
9	G20000277	ERR15948755	SAMEA120533122	1313	ftp.sra.ebi.ac.uk/vol1/fastq/ERR159/055/ERR15948755/ERR15948755_1.fastq.gz;ftp.sra.ebi.ac.uk/vol1/fastq/ERR159/055/ERR15948755/ERR15948755_2.fastq.gz
10	G20000267	ERR15948761	SAMEA120533116	1313	ftp.sra.ebi.ac.uk/vol1/fastq/ERR159/061/ERR15948761/ERR15948761_1.fastq.gz;ftp.sra.ebi.ac.uk/vol1/fastq/ERR159/061/ERR15948761/ERR15948761_2.fastq.gz
11	G20000253	ERR15948768	SAMEA120533109	1313	ftp.sra.ebi.ac.uk/vol1/fastq/ERR159/068/ERR15948768/ERR15948768_1.fastq.gz;ftp.sra.ebi.ac.uk/vol1/fastq/ERR159/068/ERR15948768/ERR15948768_2.fastq.gz
12	G18004026	ERR15948796	SAMEA120533081	1313	ftp.sra.ebi.ac.uk/vol1/fastq/ERR159/096/ERR15948796/ERR15948796_1.fastq.gz;ftp.sra.ebi.ac.uk/vol1/fastq/ERR159/096/ERR15948796/ERR15948796_2.fastq.gz
13	G18004024	ERR15948798	SAMEA120533079	1313	ftp.sra.ebi.ac.uk/vol1/fastq/ERR159/098/ERR15948798/ERR15948798_1.fastq.gz;ftp.sra.ebi.ac.uk/vol1/fastq/ERR159/098/ERR15948798/ERR15948798_2.fastq.gz
14	G18004008	ERR15948805	SAMEA120533072	1313	ftp.sra.ebi.ac.uk/vol1/fastq/ERR159/005/ERR15948805/ERR15948805_1.fastq.gz;ftp.sra.ebi.ac.uk/vol1/fastq/ERR159/005/ERR15948805/ERR15948805_2.fastq.gz
15	G18003992	ERR15948809	SAMEA120533068	1313	ftp.sra.ebi.ac.uk/vol1/fastq/ERR159/009/ERR15948809/ERR15948809_1.fastq.gz;ftp.sra.ebi.ac.uk/vol1/fastq/ERR159/009/ERR15948809/ERR15948809_2.fastq.gz
16	G18003936	ERR15948817	SAMEA120533060	1313	ftp.sra.ebi.ac.uk/vol1/fastq/ERR159/017/ERR15948817/ERR15948817_1.fastq.gz;ftp.sra.ebi.ac.uk/vol1/fastq/ERR159/017/ERR15948817/ERR15948817_2.fastq.gz
17	G18003926	ERR15948818	SAMEA120533059	1313	ftp.sra.ebi.ac.uk/vol1/fastq/ERR159/018/ERR15948818/ERR15948818_1.fastq.gz;ftp.sra.ebi.ac.uk/vol1/fastq/ERR159/018/ERR15948818/ERR15948818_2.fastq.gz
18	G18003925	ERR15948819	SAMEA120533058	1313	ftp.sra.ebi.ac.uk/vol1/fastq/ERR159/019/ERR15948819/ERR15948819_1.fastq.gz;ftp.sra.ebi.ac.uk/vol1/fastq/ERR159/019/ERR15948819/ERR15948819_2.fastq.gz
19	G18003919	ERR15948821	SAMEA120533056	1313	ftp.sra.ebi.ac.uk/vol1/fastq/ERR159/021/ERR15948821/ERR15948821_1.fastq.gz;ftp.sra.ebi.ac.uk/vol1/fastq/ERR159/021/ERR15948821/ERR15948821_2.fastq.gz
20	G18003915	ERR15948822	SAMEA120533055	1313	ftp.sra.ebi.ac.uk/vol1/fastq/ERR159/022/ERR15948822/ERR15948822_1.fastq.gz;ftp.sra.ebi.ac.uk/vol1/fastq/ERR159/022/ERR15948822/ERR15948822_2.fastq.gz

21	G18003904	ERR15948829	SAMEA120533048	1313	ftp.sra.ebi.ac.uk/vol1/fastq/ERR159/029/ERR15948829/ERR15948829_1.fastq.gz;ftp.sra.ebi.ac.uk/vol1/fastq/ERR159/029/ERR15948829/ERR15948829_2.fastq.gz
22	G18003903	ERR15948830	SAMEA120533047	1313	ftp.sra.ebi.ac.uk/vol1/fastq/ERR159/030/ERR15948830/ERR15948830_1.fastq.gz;ftp.sra.ebi.ac.uk/vol1/fastq/ERR159/030/ERR15948830/ERR15948830_2.fastq.gz
23	G18003888	ERR15948833	SAMEA120533044	1313	ftp.sra.ebi.ac.uk/vol1/fastq/ERR159/033/ERR15948833/ERR15948833_1.fastq.gz;ftp.sra.ebi.ac.uk/vol1/fastq/ERR159/033/ERR15948833/ERR15948833_2.fastq.gz
24	G18003873	ERR15948836	SAMEA120533041	1313	ftp.sra.ebi.ac.uk/vol1/fastq/ERR159/036/ERR15948836/ERR15948836_1.fastq.gz;ftp.sra.ebi.ac.uk/vol1/fastq/ERR159/036/ERR15948836/ERR15948836_2.fastq.gz
25	G18003865	ERR15948838	SAMEA120533039	1313	ftp.sra.ebi.ac.uk/vol1/fastq/ERR159/038/ERR15948838/ERR15948838_1.fastq.gz;ftp.sra.ebi.ac.uk/vol1/fastq/ERR159/038/ERR15948838/ERR15948838_2.fastq.gz
26	G18003861	ERR15948839	SAMEA120533038	1313	ftp.sra.ebi.ac.uk/vol1/fastq/ERR159/039/ERR15948839/ERR15948839_1.fastq.gz;ftp.sra.ebi.ac.uk/vol1/fastq/ERR159/039/ERR15948839/ERR15948839_2.fastq.gz
27	G18003839	ERR15948842	SAMEA120533035	1313	ftp.sra.ebi.ac.uk/vol1/fastq/ERR159/042/ERR15948842/ERR15948842_1.fastq.gz;ftp.sra.ebi.ac.uk/vol1/fastq/ERR159/042/ERR15948842/ERR15948842_2.fastq.gz
28	G18003826	ERR15948844	SAMEA120533033	1313	ftp.sra.ebi.ac.uk/vol1/fastq/ERR159/044/ERR15948844/ERR15948844_1.fastq.gz;ftp.sra.ebi.ac.uk/vol1/fastq/ERR159/044/ERR15948844/ERR15948844_2.fastq.gz
29	G18003819	ERR15948847	SAMEA120533030	1313	ftp.sra.ebi.ac.uk/vol1/fastq/ERR159/047/ERR15948847/ERR15948847_1.fastq.gz;ftp.sra.ebi.ac.uk/vol1/fastq/ERR159/047/ERR15948847/ERR15948847_2.fastq.gz
30	G18003816	ERR15948848	SAMEA120533029	1313	ftp.sra.ebi.ac.uk/vol1/fastq/ERR159/048/ERR15948848/ERR15948848_1.fastq.gz;ftp.sra.ebi.ac.uk/vol1/fastq/ERR159/048/ERR15948848/ERR15948848_2.fastq.gz
31	G18003814	ERR15948850	SAMEA120533027	1313	ftp.sra.ebi.ac.uk/vol1/fastq/ERR159/050/ERR15948850/ERR15948850_1.fastq.gz;ftp.sra.ebi.ac.uk/vol1/fastq/ERR159/050/ERR15948850/ERR15948850_2.fastq.gz
32	G18003801	ERR15948854	SAMEA120533023	1313	ftp.sra.ebi.ac.uk/vol1/fastq/ERR159/054/ERR15948854/ERR15948854_1.fastq.gz;ftp.sra.ebi.ac.uk/vol1/fastq/ERR159/054/ERR15948854/ERR15948854_2.fastq.gz
33	G18003795	ERR15948857	SAMEA120533020	1313	ftp.sra.ebi.ac.uk/vol1/fastq/ERR159/057/ERR15948857/ERR15948857_1.fastq.gz;ftp.sra.ebi.ac.uk/vol1/fastq/ERR159/057/ERR15948857/ERR15948857_2.fastq.gz
34	G18003789	ERR15948860	SAMEA120533017	1313	ftp.sra.ebi.ac.uk/vol1/fastq/ERR159/060/ERR15948860/ERR15948860_1.fastq.gz;ftp.sra.ebi.ac.uk/vol1/fastq/ERR159/060/ERR15948860/ERR15948860_2.fastq.gz
35	G18003768	ERR15948864	SAMEA120533013	1313	ftp.sra.ebi.ac.uk/vol1/fastq/ERR159/064/ERR15948864/ERR15948864_1.fastq.gz;ftp.sra.ebi.ac.uk/vol1/fastq/ERR159/064/ERR15948864/ERR15948864_2.fastq.gz
36	G18003767	ERR15948865	SAMEA120533012	1313	ftp.sra.ebi.ac.uk/vol1/fastq/ERR159/065/ERR15948865/ERR15948865_1.fastq.gz;ftp.sra.ebi.ac.uk/vol1/fastq/ERR159/065/ERR15948865/ERR15948865_2.fastq.gz
37	G18003705	ERR15948867	SAMEA120533010	1313	ftp.sra.ebi.ac.uk/vol1/fastq/ERR159/067/ERR15948867/ERR15948867_1.fastq.gz;ftp.sra.ebi.ac.uk/vol1/fastq/ERR159/067/ERR15948867/ERR15948867_2.fastq.gz
38	G18003704	ERR15948868	SAMEA120533009	1313	ftp.sra.ebi.ac.uk/vol1/fastq/ERR159/068/ERR15948868/ERR15948868_1.fastq.gz;ftp.sra.ebi.ac.uk/vol1/fastq/ERR159/068/ERR15948868/ERR15948868_2.fastq.gz
39	G18003697	ERR15948870	SAMEA120533007	1313	ftp.sra.ebi.ac.uk/vol1/fastq/ERR159/070/ERR15948870/ERR15948870_1.fastq.gz;ftp.sra.ebi.ac.uk/vol1/fastq/ERR159/070/ERR15948870/ERR15948870_2.fastq.gz
40	G18003679	ERR15948875	SAMEA120533002	1313	ftp.sra.ebi.ac.uk/vol1/fastq/ERR159/075/ERR15948875/ERR15948875_1.fastq.gz;ftp.sra.ebi.ac.uk/vol1/fastq/ERR159/075/ERR15948875/ERR15948875_2.fastq.gz
41	G18003676	ERR15948876	SAMEA120533001	1313	ftp.sra.ebi.ac.uk/vol1/fastq/ERR159/076/ERR15948876/ERR15948876_1.fastq.gz;ftp.sra.ebi.ac.uk/vol1/fastq/ERR159/076/ERR15948876/ERR15948876_2.fastq.gz

42	G18003673	ERR15948878	SAMEA120532999	1313	ftp.sra.ebi.ac.uk/vol1/fastq/ERR159/078/ERR15948878/ERR15948878_1.fastq.gz;ftp.sra.ebi.ac.uk/vol1/fastq/ERR159/078/ERR15948878/ERR15948878_2.fastq.gz
43	G18003581	ERR15948885	SAMEA120532992	1313	ftp.sra.ebi.ac.uk/vol1/fastq/ERR159/085/ERR15948885/ERR15948885_1.fastq.gz;ftp.sra.ebi.ac.uk/vol1/fastq/ERR159/085/ERR15948885/ERR15948885_2.fastq.gz
44	G18003578	ERR15948886	SAMEA120532991	1313	ftp.sra.ebi.ac.uk/vol1/fastq/ERR159/086/ERR15948886/ERR15948886_1.fastq.gz;ftp.sra.ebi.ac.uk/vol1/fastq/ERR159/086/ERR15948886/ERR15948886_2.fastq.gz
45	G18003568	ERR15948889	SAMEA120532988	1313	ftp.sra.ebi.ac.uk/vol1/fastq/ERR159/089/ERR15948889/ERR15948889_1.fastq.gz;ftp.sra.ebi.ac.uk/vol1/fastq/ERR159/089/ERR15948889/ERR15948889_2.fastq.gz
46	G18003566	ERR15948891	SAMEA120532986	1313	ftp.sra.ebi.ac.uk/vol1/fastq/ERR159/091/ERR15948891/ERR15948891_1.fastq.gz;ftp.sra.ebi.ac.uk/vol1/fastq/ERR159/091/ERR15948891/ERR15948891_2.fastq.gz
47	G18003558	ERR15948893	SAMEA120532984	1313	ftp.sra.ebi.ac.uk/vol1/fastq/ERR159/093/ERR15948893/ERR15948893_1.fastq.gz;ftp.sra.ebi.ac.uk/vol1/fastq/ERR159/093/ERR15948893/ERR15948893_2.fastq.gz
48	G18003553	ERR15948896	SAMEA120532981	1313	ftp.sra.ebi.ac.uk/vol1/fastq/ERR159/096/ERR15948896/ERR15948896_1.fastq.gz;ftp.sra.ebi.ac.uk/vol1/fastq/ERR159/096/ERR15948896/ERR15948896_2.fastq.gz
49	G18003539	ERR15948899	SAMEA120532978	1313	ftp.sra.ebi.ac.uk/vol1/fastq/ERR159/099/ERR15948899/ERR15948899_1.fastq.gz;ftp.sra.ebi.ac.uk/vol1/fastq/ERR159/099/ERR15948899/ERR15948899_2.fastq.gz
50	G18003450	ERR15948902	SAMEA120532948	1313	ftp.sra.ebi.ac.uk/vol1/fastq/ERR159/002/ERR15948902/ERR15948902_1.fastq.gz;ftp.sra.ebi.ac.uk/vol1/fastq/ERR159/002/ERR15948902/ERR15948902_2.fastq.gz
51	G18003434	ERR15948906	SAMEA120532944	1313	ftp.sra.ebi.ac.uk/vol1/fastq/ERR159/006/ERR15948906/ERR15948906_1.fastq.gz;ftp.sra.ebi.ac.uk/vol1/fastq/ERR159/006/ERR15948906/ERR15948906_2.fastq.gz
52	G18003427	ERR15948908	SAMEA120532942	1313	ftp.sra.ebi.ac.uk/vol1/fastq/ERR159/008/ERR15948908/ERR15948908_1.fastq.gz;ftp.sra.ebi.ac.uk/vol1/fastq/ERR159/008/ERR15948908/ERR15948908_2.fastq.gz
53	G18003420	ERR15948911	SAMEA120532939	1313	ftp.sra.ebi.ac.uk/vol1/fastq/ERR159/011/ERR15948911/ERR15948911_1.fastq.gz;ftp.sra.ebi.ac.uk/vol1/fastq/ERR159/011/ERR15948911/ERR15948911_2.fastq.gz
54	G18003395	ERR15948918	SAMEA120532932	1313	ftp.sra.ebi.ac.uk/vol1/fastq/ERR159/018/ERR15948918/ERR15948918_1.fastq.gz;ftp.sra.ebi.ac.uk/vol1/fastq/ERR159/018/ERR15948918/ERR15948918_2.fastq.gz
55	G18003371	ERR15948929	SAMEA120532921	1313	ftp.sra.ebi.ac.uk/vol1/fastq/ERR159/029/ERR15948929/ERR15948929_1.fastq.gz;ftp.sra.ebi.ac.uk/vol1/fastq/ERR159/029/ERR15948929/ERR15948929_2.fastq.gz
56	G18003370	ERR15948930	SAMEA120532920	1313	ftp.sra.ebi.ac.uk/vol1/fastq/ERR159/030/ERR15948930/ERR15948930_1.fastq.gz;ftp.sra.ebi.ac.uk/vol1/fastq/ERR159/030/ERR15948930/ERR15948930_2.fastq.gz
57	G18003364	ERR15948931	SAMEA120532919	1313	ftp.sra.ebi.ac.uk/vol1/fastq/ERR159/031/ERR15948931/ERR15948931_1.fastq.gz;ftp.sra.ebi.ac.uk/vol1/fastq/ERR159/031/ERR15948931/ERR15948931_2.fastq.gz
58	G18003354	ERR15948934	SAMEA120532916	1313	ftp.sra.ebi.ac.uk/vol1/fastq/ERR159/034/ERR15948934/ERR15948934_1.fastq.gz;ftp.sra.ebi.ac.uk/vol1/fastq/ERR159/034/ERR15948934/ERR15948934_2.fastq.gz
59	G18003340	ERR15948936	SAMEA120532914	1313	ftp.sra.ebi.ac.uk/vol1/fastq/ERR159/036/ERR15948936/ERR15948936_1.fastq.gz;ftp.sra.ebi.ac.uk/vol1/fastq/ERR159/036/ERR15948936/ERR15948936_2.fastq.gz
60	G18003325	ERR15948941	SAMEA120532909	1313	ftp.sra.ebi.ac.uk/vol1/fastq/ERR159/041/ERR15948941/ERR15948941_1.fastq.gz;ftp.sra.ebi.ac.uk/vol1/fastq/ERR159/041/ERR15948941/ERR15948941_2.fastq.gz
61	G18003322	ERR15948943	SAMEA120532907	1313	ftp.sra.ebi.ac.uk/vol1/fastq/ERR159/043/ERR15948943/ERR15948943_1.fastq.gz;ftp.sra.ebi.ac.uk/vol1/fastq/ERR159/043/ERR15948943/ERR15948943_2.fastq.gz
62	G18003321	ERR15948944	SAMEA120532906	1313	ftp.sra.ebi.ac.uk/vol1/fastq/ERR159/044/ERR15948944/ERR15948944_1.fastq.gz;ftp.sra.ebi.ac.uk/vol1/fastq/ERR159/044/ERR15948944/ERR15948944_2.fastq.gz

63	G18003319	ERR15948945	SAMEA120532905	1313	ftp.sra.ebi.ac.uk/vol1/fastq/ERR159/045/ERR15948945/ERR15948945_1.fastq.gz;ftp.sra.ebi.ac.uk/vol1/fastq/ERR159/045/ERR15948945/ERR15948945_2.fastq.gz
64	G18003316	ERR15948947	SAMEA120532903	1313	ftp.sra.ebi.ac.uk/vol1/fastq/ERR159/047/ERR15948947/ERR15948947_1.fastq.gz;ftp.sra.ebi.ac.uk/vol1/fastq/ERR159/047/ERR15948947/ERR15948947_2.fastq.gz
65	G18003307	ERR15948952	SAMEA120532898	1313	ftp.sra.ebi.ac.uk/vol1/fastq/ERR159/052/ERR15948952/ERR15948952_1.fastq.gz;ftp.sra.ebi.ac.uk/vol1/fastq/ERR159/052/ERR15948952/ERR15948952_2.fastq.gz
66	G18003289	ERR15948954	SAMEA120532896	1313	ftp.sra.ebi.ac.uk/vol1/fastq/ERR159/054/ERR15948954/ERR15948954_1.fastq.gz;ftp.sra.ebi.ac.uk/vol1/fastq/ERR159/054/ERR15948954/ERR15948954_2.fastq.gz
67	G18003283	ERR15948957	SAMEA120532893	1313	ftp.sra.ebi.ac.uk/vol1/fastq/ERR159/057/ERR15948957/ERR15948957_1.fastq.gz;ftp.sra.ebi.ac.uk/vol1/fastq/ERR159/057/ERR15948957/ERR15948957_2.fastq.gz
68	G18003272	ERR15948962	SAMEA120532888	1313	ftp.sra.ebi.ac.uk/vol1/fastq/ERR159/062/ERR15948962/ERR15948962_1.fastq.gz;ftp.sra.ebi.ac.uk/vol1/fastq/ERR159/062/ERR15948962/ERR15948962_2.fastq.gz
69	G18003263	ERR15948964	SAMEA120532886	1313	ftp.sra.ebi.ac.uk/vol1/fastq/ERR159/064/ERR15948964/ERR15948964_1.fastq.gz;ftp.sra.ebi.ac.uk/vol1/fastq/ERR159/064/ERR15948964/ERR15948964_2.fastq.gz
70	G18003260	ERR15948965	SAMEA120532885	1313	ftp.sra.ebi.ac.uk/vol1/fastq/ERR159/065/ERR15948965/ERR15948965_1.fastq.gz;ftp.sra.ebi.ac.uk/vol1/fastq/ERR159/065/ERR15948965/ERR15948965_2.fastq.gz
71	G18003251	ERR15948971	SAMEA120532879	1313	ftp.sra.ebi.ac.uk/vol1/fastq/ERR159/071/ERR15948971/ERR15948971_1.fastq.gz;ftp.sra.ebi.ac.uk/vol1/fastq/ERR159/071/ERR15948971/ERR15948971_2.fastq.gz
72	G18003250	ERR15948972	SAMEA120532878	1313	ftp.sra.ebi.ac.uk/vol1/fastq/ERR159/072/ERR15948972/ERR15948972_1.fastq.gz;ftp.sra.ebi.ac.uk/vol1/fastq/ERR159/072/ERR15948972/ERR15948972_2.fastq.gz
73	G18003248	ERR15948974	SAMEA120532876	1313	ftp.sra.ebi.ac.uk/vol1/fastq/ERR159/074/ERR15948974/ERR15948974_1.fastq.gz;ftp.sra.ebi.ac.uk/vol1/fastq/ERR159/074/ERR15948974/ERR15948974_2.fastq.gz
74	G18003245	ERR15948977	SAMEA120532873	1313	ftp.sra.ebi.ac.uk/vol1/fastq/ERR159/077/ERR15948977/ERR15948977_1.fastq.gz;ftp.sra.ebi.ac.uk/vol1/fastq/ERR159/077/ERR15948977/ERR15948977_2.fastq.gz
75	G18003531	ERR15948978	SAMEA120532976	1313	ftp.sra.ebi.ac.uk/vol1/fastq/ERR159/078/ERR15948978/ERR15948978_1.fastq.gz;ftp.sra.ebi.ac.uk/vol1/fastq/ERR159/078/ERR15948978/ERR15948978_2.fastq.gz
76	G18003518	ERR15948985	SAMEA120532969	1313	ftp.sra.ebi.ac.uk/vol1/fastq/ERR159/085/ERR15948985/ERR15948985_1.fastq.gz;ftp.sra.ebi.ac.uk/vol1/fastq/ERR159/085/ERR15948985/ERR15948985_2.fastq.gz
77	G18003517	ERR15948986	SAMEA120532968	1313	ftp.sra.ebi.ac.uk/vol1/fastq/ERR159/086/ERR15948986/ERR15948986_1.fastq.gz;ftp.sra.ebi.ac.uk/vol1/fastq/ERR159/086/ERR15948986/ERR15948986_2.fastq.gz
78	G18003496	ERR15948992	SAMEA120532962	1313	ftp.sra.ebi.ac.uk/vol1/fastq/ERR159/092/ERR15948992/ERR15948992_1.fastq.gz;ftp.sra.ebi.ac.uk/vol1/fastq/ERR159/092/ERR15948992/ERR15948992_2.fastq.gz
79	G18003494	ERR15948993	SAMEA120532961	1313	ftp.sra.ebi.ac.uk/vol1/fastq/ERR159/093/ERR15948993/ERR15948993_1.fastq.gz;ftp.sra.ebi.ac.uk/vol1/fastq/ERR159/093/ERR15948993/ERR15948993_2.fastq.gz
80	G18003493	ERR15948994	SAMEA120532960	1313	ftp.sra.ebi.ac.uk/vol1/fastq/ERR159/094/ERR15948994/ERR15948994_1.fastq.gz;ftp.sra.ebi.ac.uk/vol1/fastq/ERR159/094/ERR15948994/ERR15948994_2.fastq.gz
81	G18003476	ERR15948998	SAMEA120532956	1313	ftp.sra.ebi.ac.uk/vol1/fastq/ERR159/098/ERR15948998/ERR15948998_1.fastq.gz;ftp.sra.ebi.ac.uk/vol1/fastq/ERR159/098/ERR15948998/ERR15948998_2.fastq.gz
82	G18003460	ERR15949003	SAMEA120532951	1313	ftp.sra.ebi.ac.uk/vol1/fastq/ERR159/003/ERR15949003/ERR15949003_1.fastq.gz;ftp.sra.ebi.ac.uk/vol1/fastq/ERR159/003/ERR15949003/ERR15949003_2.fastq.gz
83	G20000372	ERR15948698	SAMEA120533179	1313	ftp.sra.ebi.ac.uk/vol1/fastq/ERR159/098/ERR15948698/ERR15948698_1.fastq.gz;ftp.sra.ebi.ac.uk/vol1/fastq/ERR159/098/ERR15948698/ERR15948698_2.fastq.gz

84	G20000361	ERR15948703	SAMEA120533174	1313	ftp.sra.ebi.ac.uk/vol1/fastq/ERR159/003/ERR15948703/ERR15948703_1.fastq.gz;ftp.sra.ebi.ac.uk/vol1/fastq/ERR159/003/ERR15948703/ERR15948703_2.fastq.gz
85	G20000353	ERR15948708	SAMEA120533169	1313	ftp.sra.ebi.ac.uk/vol1/fastq/ERR159/008/ERR15948708/ERR15948708_1.fastq.gz;ftp.sra.ebi.ac.uk/vol1/fastq/ERR159/008/ERR15948708/ERR15948708_2.fastq.gz
86	G20000344	ERR15948713	SAMEA120533164	1313	ftp.sra.ebi.ac.uk/vol1/fastq/ERR159/013/ERR15948713/ERR15948713_1.fastq.gz;ftp.sra.ebi.ac.uk/vol1/fastq/ERR159/013/ERR15948713/ERR15948713_2.fastq.gz
87	G20000325	ERR15948725	SAMEA120533152	1313	ftp.sra.ebi.ac.uk/vol1/fastq/ERR159/025/ERR15948725/ERR15948725_1.fastq.gz;ftp.sra.ebi.ac.uk/vol1/fastq/ERR159/025/ERR15948725/ERR15948725_2.fastq.gz
88	G20000321	ERR15948728	SAMEA120533149	1313	ftp.sra.ebi.ac.uk/vol1/fastq/ERR159/028/ERR15948728/ERR15948728_1.fastq.gz;ftp.sra.ebi.ac.uk/vol1/fastq/ERR159/028/ERR15948728/ERR15948728_2.fastq.gz
89	G20000318	ERR15948729	SAMEA120533148	1313	ftp.sra.ebi.ac.uk/vol1/fastq/ERR159/029/ERR15948729/ERR15948729_1.fastq.gz;ftp.sra.ebi.ac.uk/vol1/fastq/ERR159/029/ERR15948729/ERR15948729_2.fastq.gz
90	G20000314	ERR15948731	SAMEA120533146	1313	ftp.sra.ebi.ac.uk/vol1/fastq/ERR159/031/ERR15948731/ERR15948731_1.fastq.gz;ftp.sra.ebi.ac.uk/vol1/fastq/ERR159/031/ERR15948731/ERR15948731_2.fastq.gz
91	G20000311	ERR15948734	SAMEA120533143	1313	ftp.sra.ebi.ac.uk/vol1/fastq/ERR159/034/ERR15948734/ERR15948734_1.fastq.gz;ftp.sra.ebi.ac.uk/vol1/fastq/ERR159/034/ERR15948734/ERR15948734_2.fastq.gz
92	G20000301	ERR15948738	SAMEA120533139	1313	ftp.sra.ebi.ac.uk/vol1/fastq/ERR159/038/ERR15948738/ERR15948738_1.fastq.gz;ftp.sra.ebi.ac.uk/vol1/fastq/ERR159/038/ERR15948738/ERR15948738_2.fastq.gz
93	G20000297	ERR15948740	SAMEA120533137	1313	ftp.sra.ebi.ac.uk/vol1/fastq/ERR159/040/ERR15948740/ERR15948740_1.fastq.gz;ftp.sra.ebi.ac.uk/vol1/fastq/ERR159/040/ERR15948740/ERR15948740_2.fastq.gz
94	G20000291	ERR15948745	SAMEA120533132	1313	ftp.sra.ebi.ac.uk/vol1/fastq/ERR159/045/ERR15948745/ERR15948745_1.fastq.gz;ftp.sra.ebi.ac.uk/vol1/fastq/ERR159/045/ERR15948745/ERR15948745_2.fastq.gz
95	G20000289	ERR15948747	SAMEA120533130	1313	ftp.sra.ebi.ac.uk/vol1/fastq/ERR159/047/ERR15948747/ERR15948747_1.fastq.gz;ftp.sra.ebi.ac.uk/vol1/fastq/ERR159/047/ERR15948747/ERR15948747_2.fastq.gz
96	G20000280	ERR15948752	SAMEA120533125	1313	ftp.sra.ebi.ac.uk/vol1/fastq/ERR159/052/ERR15948752/ERR15948752_1.fastq.gz;ftp.sra.ebi.ac.uk/vol1/fastq/ERR159/052/ERR15948752/ERR15948752_2.fastq.gz
97	G20000279	ERR15948753	SAMEA120533124	1313	ftp.sra.ebi.ac.uk/vol1/fastq/ERR159/053/ERR15948753/ERR15948753_1.fastq.gz;ftp.sra.ebi.ac.uk/vol1/fastq/ERR159/053/ERR15948753/ERR15948753_2.fastq.gz
98	G20000278	ERR15948754	SAMEA120533123	1313	ftp.sra.ebi.ac.uk/vol1/fastq/ERR159/054/ERR15948754/ERR15948754_1.fastq.gz;ftp.sra.ebi.ac.uk/vol1/fastq/ERR159/054/ERR15948754/ERR15948754_2.fastq.gz
99	G20000272	ERR15948758	SAMEA120533119	1313	ftp.sra.ebi.ac.uk/vol1/fastq/ERR159/058/ERR15948758/ERR15948758_1.fastq.gz;ftp.sra.ebi.ac.uk/vol1/fastq/ERR159/058/ERR15948758/ERR15948758_2.fastq.gz
100	G20000268	ERR15948760	SAMEA120533117	1313	ftp.sra.ebi.ac.uk/vol1/fastq/ERR159/060/ERR15948760/ERR15948760_1.fastq.gz;ftp.sra.ebi.ac.uk/vol1/fastq/ERR159/060/ERR15948760/ERR15948760_2.fastq.gz
101	G20000266	ERR15948762	SAMEA120533115	1313	ftp.sra.ebi.ac.uk/vol1/fastq/ERR159/062/ERR15948762/ERR15948762_1.fastq.gz;ftp.sra.ebi.ac.uk/vol1/fastq/ERR159/062/ERR15948762/ERR15948762_2.fastq.gz
102	G20000259	ERR15948765	SAMEA120533112	1313	ftp.sra.ebi.ac.uk/vol1/fastq/ERR159/065/ERR15948765/ERR15948765_1.fastq.gz;ftp.sra.ebi.ac.uk/vol1/fastq/ERR159/065/ERR15948765/ERR15948765_2.fastq.gz
103	G20000238	ERR15948774	SAMEA120533103	1313	ftp.sra.ebi.ac.uk/vol1/fastq/ERR159/074/ERR15948774/ERR15948774_1.fastq.gz;ftp.sra.ebi.ac.uk/vol1/fastq/ERR159/074/ERR15948774/ERR15948774_2.fastq.gz
104	G20000237	ERR15948775	SAMEA120533102	1313	ftp.sra.ebi.ac.uk/vol1/fastq/ERR159/075/ERR15948775/ERR15948775_1.fastq.gz;ftp.sra.ebi.ac.uk/vol1/fastq/ERR159/075/ERR15948775/ERR15948775_2.fastq.gz

105	G20000230	ERR15948779	SAMEA120533098	1313	ftp.sra.ebi.ac.uk/vol1/fastq/ERR159/079/ERR15948779/ERR15948779_1.fastq.gz;ftp.sra.ebi.ac.uk/vol1/fastq/ERR159/079/ERR15948779/ERR15948779_2.fastq.gz
106	G20000221	ERR15948784	SAMEA120533093	1313	ftp.sra.ebi.ac.uk/vol1/fastq/ERR159/084/ERR15948784/ERR15948784_1.fastq.gz;ftp.sra.ebi.ac.uk/vol1/fastq/ERR159/084/ERR15948784/ERR15948784_2.fastq.gz
107	G20000210	ERR15948790	SAMEA120533087	1313	ftp.sra.ebi.ac.uk/vol1/fastq/ERR159/090/ERR15948790/ERR15948790_1.fastq.gz;ftp.sra.ebi.ac.uk/vol1/fastq/ERR159/090/ERR15948790/ERR15948790_2.fastq.gz
108	G20000202	ERR15948795	SAMEA120533082	1313	ftp.sra.ebi.ac.uk/vol1/fastq/ERR159/095/ERR15948795/ERR15948795_1.fastq.gz;ftp.sra.ebi.ac.uk/vol1/fastq/ERR159/095/ERR15948795/ERR15948795_2.fastq.gz
109	G18003404	ERR15948916	SAMEA120532934	1313	ftp.sra.ebi.ac.uk/vol1/fastq/ERR159/016/ERR15948916/ERR15948916_1.fastq.gz;ftp.sra.ebi.ac.uk/vol1/fastq/ERR159/016/ERR15948916/ERR15948916_2.fastq.gz
110	G20000385	ERR15948692	SAMEA120533185	1313	ftp.sra.ebi.ac.uk/vol1/fastq/ERR159/092/ERR15948692/ERR15948692_1.fastq.gz;ftp.sra.ebi.ac.uk/vol1/fastq/ERR159/092/ERR15948692/ERR15948692_2.fastq.gz
111	G20000375	ERR15948696	SAMEA120533181	1313	ftp.sra.ebi.ac.uk/vol1/fastq/ERR159/096/ERR15948696/ERR15948696_1.fastq.gz;ftp.sra.ebi.ac.uk/vol1/fastq/ERR159/096/ERR15948696/ERR15948696_2.fastq.gz
112	G20000359	ERR15948705	SAMEA120533172	1313	ftp.sra.ebi.ac.uk/vol1/fastq/ERR159/005/ERR15948705/ERR15948705_1.fastq.gz;ftp.sra.ebi.ac.uk/vol1/fastq/ERR159/005/ERR15948705/ERR15948705_2.fastq.gz
113	G20000348	ERR15948709	SAMEA120533168	1313	ftp.sra.ebi.ac.uk/vol1/fastq/ERR159/009/ERR15948709/ERR15948709_1.fastq.gz;ftp.sra.ebi.ac.uk/vol1/fastq/ERR159/009/ERR15948709/ERR15948709_2.fastq.gz
114	G20000343	ERR15948714	SAMEA120533163	1313	ftp.sra.ebi.ac.uk/vol1/fastq/ERR159/014/ERR15948714/ERR15948714_1.fastq.gz;ftp.sra.ebi.ac.uk/vol1/fastq/ERR159/014/ERR15948714/ERR15948714_2.fastq.gz
115	G20000290	ERR15948746	SAMEA120533131	1313	ftp.sra.ebi.ac.uk/vol1/fastq/ERR159/046/ERR15948746/ERR15948746_1.fastq.gz;ftp.sra.ebi.ac.uk/vol1/fastq/ERR159/046/ERR15948746/ERR15948746_2.fastq.gz
116	G20000276	ERR15948756	SAMEA120533121	1313	ftp.sra.ebi.ac.uk/vol1/fastq/ERR159/056/ERR15948756/ERR15948756_1.fastq.gz;ftp.sra.ebi.ac.uk/vol1/fastq/ERR159/056/ERR15948756/ERR15948756_2.fastq.gz
117	G20000254	ERR15948767	SAMEA120533110	1313	ftp.sra.ebi.ac.uk/vol1/fastq/ERR159/067/ERR15948767/ERR15948767_1.fastq.gz;ftp.sra.ebi.ac.uk/vol1/fastq/ERR159/067/ERR15948767/ERR15948767_2.fastq.gz
118	G20000239	ERR15948773	SAMEA120533104	1313	ftp.sra.ebi.ac.uk/vol1/fastq/ERR159/073/ERR15948773/ERR15948773_1.fastq.gz;ftp.sra.ebi.ac.uk/vol1/fastq/ERR159/073/ERR15948773/ERR15948773_2.fastq.gz
119	G20000234	ERR15948776	SAMEA120533101	1313	ftp.sra.ebi.ac.uk/vol1/fastq/ERR159/076/ERR15948776/ERR15948776_1.fastq.gz;ftp.sra.ebi.ac.uk/vol1/fastq/ERR159/076/ERR15948776/ERR15948776_2.fastq.gz
120	G20000231	ERR15948778	SAMEA120533099	1313	ftp.sra.ebi.ac.uk/vol1/fastq/ERR159/078/ERR15948778/ERR15948778_1.fastq.gz;ftp.sra.ebi.ac.uk/vol1/fastq/ERR159/078/ERR15948778/ERR15948778_2.fastq.gz
121	G20000228	ERR15948781	SAMEA120533096	1313	ftp.sra.ebi.ac.uk/vol1/fastq/ERR159/081/ERR15948781/ERR15948781_1.fastq.gz;ftp.sra.ebi.ac.uk/vol1/fastq/ERR159/081/ERR15948781/ERR15948781_2.fastq.gz
122	G20000224	ERR15948782	SAMEA120533095	1313	ftp.sra.ebi.ac.uk/vol1/fastq/ERR159/082/ERR15948782/ERR15948782_1.fastq.gz;ftp.sra.ebi.ac.uk/vol1/fastq/ERR159/082/ERR15948782/ERR15948782_2.fastq.gz
123	G20000211	ERR15948789	SAMEA120533088	1313	ftp.sra.ebi.ac.uk/vol1/fastq/ERR159/089/ERR15948789/ERR15948789_1.fastq.gz;ftp.sra.ebi.ac.uk/vol1/fastq/ERR159/089/ERR15948789/ERR15948789_2.fastq.gz
124	G20000206	ERR15948792	SAMEA120533085	1313	ftp.sra.ebi.ac.uk/vol1/fastq/ERR159/092/ERR15948792/ERR15948792_1.fastq.gz;ftp.sra.ebi.ac.uk/vol1/fastq/ERR159/092/ERR15948792/ERR15948792_2.fastq.gz
125	G20000204	ERR15948793	SAMEA120533084	1313	ftp.sra.ebi.ac.uk/vol1/fastq/ERR159/093/ERR15948793/ERR15948793_1.fastq.gz;ftp.sra.ebi.ac.uk/vol1/fastq/ERR159/093/ERR15948793/ERR15948793_2.fastq.gz

126	G18004023	ERR15948799	SAMEA120533078	1313	ftp.sra.ebi.ac.uk/vol1/fastq/ERR159/099/ERR15948799/ERR15948799_1.fastq.gz;ftp.sra.ebi.ac.uk/vol1/fastq/ERR159/099/ERR15948799/ERR15948799_2.fastq.gz
127	G18004009	ERR15948804	SAMEA120533073	1313	ftp.sra.ebi.ac.uk/vol1/fastq/ERR159/004/ERR15948804/ERR15948804_1.fastq.gz;ftp.sra.ebi.ac.uk/vol1/fastq/ERR159/004/ERR15948804/ERR15948804_2.fastq.gz
128	G18004000	ERR15948807	SAMEA120533070	1313	ftp.sra.ebi.ac.uk/vol1/fastq/ERR159/007/ERR15948807/ERR15948807_1.fastq.gz;ftp.sra.ebi.ac.uk/vol1/fastq/ERR159/007/ERR15948807/ERR15948807_2.fastq.gz
129	G18003999	ERR15948808	SAMEA120533069	1313	ftp.sra.ebi.ac.uk/vol1/fastq/ERR159/008/ERR15948808/ERR15948808_1.fastq.gz;ftp.sra.ebi.ac.uk/vol1/fastq/ERR159/008/ERR15948808/ERR15948808_2.fastq.gz
130	G18003957	ERR15948812	SAMEA120533065	1313	ftp.sra.ebi.ac.uk/vol1/fastq/ERR159/012/ERR15948812/ERR15948812_1.fastq.gz;ftp.sra.ebi.ac.uk/vol1/fastq/ERR159/012/ERR15948812/ERR15948812_2.fastq.gz
131	G18003948	ERR15948815	SAMEA120533062	1313	ftp.sra.ebi.ac.uk/vol1/fastq/ERR159/015/ERR15948815/ERR15948815_1.fastq.gz;ftp.sra.ebi.ac.uk/vol1/fastq/ERR159/015/ERR15948815/ERR15948815_2.fastq.gz
132	G18003922	ERR15948820	SAMEA120533057	1313	ftp.sra.ebi.ac.uk/vol1/fastq/ERR159/020/ERR15948820/ERR15948820_1.fastq.gz;ftp.sra.ebi.ac.uk/vol1/fastq/ERR159/020/ERR15948820/ERR15948820_2.fastq.gz
133	G18003913	ERR15948823	SAMEA120533054	1313	ftp.sra.ebi.ac.uk/vol1/fastq/ERR159/023/ERR15948823/ERR15948823_1.fastq.gz;ftp.sra.ebi.ac.uk/vol1/fastq/ERR159/023/ERR15948823/ERR15948823_2.fastq.gz
134	G18003909	ERR15948825	SAMEA120533052	1313	ftp.sra.ebi.ac.uk/vol1/fastq/ERR159/025/ERR15948825/ERR15948825_1.fastq.gz;ftp.sra.ebi.ac.uk/vol1/fastq/ERR159/025/ERR15948825/ERR15948825_2.fastq.gz
135	G18003891	ERR15948832	SAMEA120533045	1313	ftp.sra.ebi.ac.uk/vol1/fastq/ERR159/032/ERR15948832/ERR15948832_1.fastq.gz;ftp.sra.ebi.ac.uk/vol1/fastq/ERR159/032/ERR15948832/ERR15948832_2.fastq.gz
136	G18003880	ERR15948835	SAMEA120533042	1313	ftp.sra.ebi.ac.uk/vol1/fastq/ERR159/035/ERR15948835/ERR15948835_1.fastq.gz;ftp.sra.ebi.ac.uk/vol1/fastq/ERR159/035/ERR15948835/ERR15948835_2.fastq.gz
137	G18003869	ERR15948837	SAMEA120533040	1313	ftp.sra.ebi.ac.uk/vol1/fastq/ERR159/037/ERR15948837/ERR15948837_1.fastq.gz;ftp.sra.ebi.ac.uk/vol1/fastq/ERR159/037/ERR15948837/ERR15948837_2.fastq.gz
138	G18003860	ERR15948840	SAMEA120533037	1313	ftp.sra.ebi.ac.uk/vol1/fastq/ERR159/040/ERR15948840/ERR15948840_1.fastq.gz;ftp.sra.ebi.ac.uk/vol1/fastq/ERR159/040/ERR15948840/ERR15948840_2.fastq.gz
139	G18003822	ERR15948845	SAMEA120533032	1313	ftp.sra.ebi.ac.uk/vol1/fastq/ERR159/045/ERR15948845/ERR15948845_1.fastq.gz;ftp.sra.ebi.ac.uk/vol1/fastq/ERR159/045/ERR15948845/ERR15948845_2.fastq.gz
140	G18003821	ERR15948846	SAMEA120533031	1313	ftp.sra.ebi.ac.uk/vol1/fastq/ERR159/046/ERR15948846/ERR15948846_1.fastq.gz;ftp.sra.ebi.ac.uk/vol1/fastq/ERR159/046/ERR15948846/ERR15948846_2.fastq.gz
141	G18003815	ERR15948849	SAMEA120533028	1313	ftp.sra.ebi.ac.uk/vol1/fastq/ERR159/049/ERR15948849/ERR15948849_1.fastq.gz;ftp.sra.ebi.ac.uk/vol1/fastq/ERR159/049/ERR15948849/ERR15948849_2.fastq.gz
142	G18003811	ERR15948851	SAMEA120533026	1313	ftp.sra.ebi.ac.uk/vol1/fastq/ERR159/051/ERR15948851/ERR15948851_1.fastq.gz;ftp.sra.ebi.ac.uk/vol1/fastq/ERR159/051/ERR15948851/ERR15948851_2.fastq.gz
143	G18003804	ERR15948852	SAMEA120533025	1313	ftp.sra.ebi.ac.uk/vol1/fastq/ERR159/052/ERR15948852/ERR15948852_1.fastq.gz;ftp.sra.ebi.ac.uk/vol1/fastq/ERR159/052/ERR15948852/ERR15948852_2.fastq.gz
144	G18003800	ERR15948855	SAMEA120533022	1313	ftp.sra.ebi.ac.uk/vol1/fastq/ERR159/055/ERR15948855/ERR15948855_1.fastq.gz;ftp.sra.ebi.ac.uk/vol1/fastq/ERR159/055/ERR15948855/ERR15948855_2.fastq.gz
145	G18003799	ERR15948856	SAMEA120533021	1313	ftp.sra.ebi.ac.uk/vol1/fastq/ERR159/056/ERR15948856/ERR15948856_1.fastq.gz;ftp.sra.ebi.ac.uk/vol1/fastq/ERR159/056/ERR15948856/ERR15948856_2.fastq.gz
146	G18003794	ERR15948858	SAMEA120533019	1313	ftp.sra.ebi.ac.uk/vol1/fastq/ERR159/058/ERR15948858/ERR15948858_1.fastq.gz;ftp.sra.ebi.ac.uk/vol1/fastq/ERR159/058/ERR15948858/ERR15948858_2.fastq.gz

147	G18003776	ERR15948861	SAMEA120533016	1313	ftp.sra.ebi.ac.uk/vol1/fastq/ERR159/061/ERR15948861/ERR15948861_1.fastq.gz;ftp.sra.ebi.ac.uk/vol1/fastq/ERR159/061/ERR15948861/ERR15948861_2.fastq.gz
148	G18003771	ERR15948862	SAMEA120533015	1313	ftp.sra.ebi.ac.uk/vol1/fastq/ERR159/062/ERR15948862/ERR15948862_1.fastq.gz;ftp.sra.ebi.ac.uk/vol1/fastq/ERR159/062/ERR15948862/ERR15948862_2.fastq.gz
149	G18003703	ERR15948869	SAMEA120533008	1313	ftp.sra.ebi.ac.uk/vol1/fastq/ERR159/069/ERR15948869/ERR15948869_1.fastq.gz;ftp.sra.ebi.ac.uk/vol1/fastq/ERR159/069/ERR15948869/ERR15948869_2.fastq.gz
150	G18003683	ERR15948874	SAMEA120533003	1313	ftp.sra.ebi.ac.uk/vol1/fastq/ERR159/074/ERR15948874/ERR15948874_1.fastq.gz;ftp.sra.ebi.ac.uk/vol1/fastq/ERR159/074/ERR15948874/ERR15948874_2.fastq.gz
151	G18003672	ERR15948879	SAMEA120532998	1313	ftp.sra.ebi.ac.uk/vol1/fastq/ERR159/079/ERR15948879/ERR15948879_1.fastq.gz;ftp.sra.ebi.ac.uk/vol1/fastq/ERR159/079/ERR15948879/ERR15948879_2.fastq.gz
152	G18003602	ERR15948881	SAMEA120532996	1313	ftp.sra.ebi.ac.uk/vol1/fastq/ERR159/081/ERR15948881/ERR15948881_1.fastq.gz;ftp.sra.ebi.ac.uk/vol1/fastq/ERR159/081/ERR15948881/ERR15948881_2.fastq.gz
153	G18003599	ERR15948883	SAMEA120532994	1313	ftp.sra.ebi.ac.uk/vol1/fastq/ERR159/083/ERR15948883/ERR15948883_1.fastq.gz;ftp.sra.ebi.ac.uk/vol1/fastq/ERR159/083/ERR15948883/ERR15948883_2.fastq.gz
154	G18003582	ERR15948884	SAMEA120532993	1313	ftp.sra.ebi.ac.uk/vol1/fastq/ERR159/084/ERR15948884/ERR15948884_1.fastq.gz;ftp.sra.ebi.ac.uk/vol1/fastq/ERR159/084/ERR15948884/ERR15948884_2.fastq.gz
155	G18003577	ERR15948887	SAMEA120532990	1313	ftp.sra.ebi.ac.uk/vol1/fastq/ERR159/087/ERR15948887/ERR15948887_1.fastq.gz;ftp.sra.ebi.ac.uk/vol1/fastq/ERR159/087/ERR15948887/ERR15948887_2.fastq.gz
156	G18003574	ERR15948888	SAMEA120532989	1313	ftp.sra.ebi.ac.uk/vol1/fastq/ERR159/088/ERR15948888/ERR15948888_1.fastq.gz;ftp.sra.ebi.ac.uk/vol1/fastq/ERR159/088/ERR15948888/ERR15948888_2.fastq.gz
157	G18003563	ERR15948892	SAMEA120532985	1313	ftp.sra.ebi.ac.uk/vol1/fastq/ERR159/092/ERR15948892/ERR15948892_1.fastq.gz;ftp.sra.ebi.ac.uk/vol1/fastq/ERR159/092/ERR15948892/ERR15948892_2.fastq.gz
158	G18003556	ERR15948895	SAMEA120532982	1313	ftp.sra.ebi.ac.uk/vol1/fastq/ERR159/095/ERR15948895/ERR15948895_1.fastq.gz;ftp.sra.ebi.ac.uk/vol1/fastq/ERR159/095/ERR15948895/ERR15948895_2.fastq.gz
159	G18003541	ERR15948897	SAMEA120532980	1313	ftp.sra.ebi.ac.uk/vol1/fastq/ERR159/097/ERR15948897/ERR15948897_1.fastq.gz;ftp.sra.ebi.ac.uk/vol1/fastq/ERR159/097/ERR15948897/ERR15948897_2.fastq.gz
160	G18003454	ERR15948901	SAMEA120532949	1313	ftp.sra.ebi.ac.uk/vol1/fastq/ERR159/001/ERR15948901/ERR15948901_1.fastq.gz;ftp.sra.ebi.ac.uk/vol1/fastq/ERR159/001/ERR15948901/ERR15948901_2.fastq.gz
161	G18003448	ERR15948903	SAMEA120532947	1313	ftp.sra.ebi.ac.uk/vol1/fastq/ERR159/003/ERR15948903/ERR15948903_1.fastq.gz;ftp.sra.ebi.ac.uk/vol1/fastq/ERR159/003/ERR15948903/ERR15948903_2.fastq.gz
162	G18003439	ERR15948904	SAMEA120532946	1313	ftp.sra.ebi.ac.uk/vol1/fastq/ERR159/004/ERR15948904/ERR15948904_1.fastq.gz;ftp.sra.ebi.ac.uk/vol1/fastq/ERR159/004/ERR15948904/ERR15948904_2.fastq.gz
163	G18003437	ERR15948905	SAMEA120532945	1313	ftp.sra.ebi.ac.uk/vol1/fastq/ERR159/005/ERR15948905/ERR15948905_1.fastq.gz;ftp.sra.ebi.ac.uk/vol1/fastq/ERR159/005/ERR15948905/ERR15948905_2.fastq.gz
164	G18003424	ERR15948909	SAMEA120532941	1313	ftp.sra.ebi.ac.uk/vol1/fastq/ERR159/009/ERR15948909/ERR15948909_1.fastq.gz;ftp.sra.ebi.ac.uk/vol1/fastq/ERR159/009/ERR15948909/ERR15948909_2.fastq.gz
165	G18003422	ERR15948910	SAMEA120532940	1313	ftp.sra.ebi.ac.uk/vol1/fastq/ERR159/010/ERR15948910/ERR15948910_1.fastq.gz;ftp.sra.ebi.ac.uk/vol1/fastq/ERR159/010/ERR15948910/ERR15948910_2.fastq.gz
166	G18003416	ERR15948913	SAMEA120532937	1313	ftp.sra.ebi.ac.uk/vol1/fastq/ERR159/013/ERR15948913/ERR15948913_1.fastq.gz;ftp.sra.ebi.ac.uk/vol1/fastq/ERR159/013/ERR15948913/ERR15948913_2.fastq.gz
167	G18003410	ERR15948915	SAMEA120532935	1313	ftp.sra.ebi.ac.uk/vol1/fastq/ERR159/015/ERR15948915/ERR15948915_1.fastq.gz;ftp.sra.ebi.ac.uk/vol1/fastq/ERR159/015/ERR15948915/ERR15948915_2.fastq.gz

168	G18003388	ERR15948920	SAMEA120532930	1313	ftp.sra.ebi.ac.uk/vol1/fastq/ERR159/020/ERR15948920/ERR15948920_1.fastq.gz;ftp.sra.ebi.ac.uk/vol1/fastq/ERR159/020/ERR15948920/ERR15948920_2.fastq.gz
169	G18003384	ERR15948921	SAMEA120532929	1313	ftp.sra.ebi.ac.uk/vol1/fastq/ERR159/021/ERR15948921/ERR15948921_1.fastq.gz;ftp.sra.ebi.ac.uk/vol1/fastq/ERR159/021/ERR15948921/ERR15948921_2.fastq.gz
170	G18003376	ERR15948925	SAMEA120532925	1313	ftp.sra.ebi.ac.uk/vol1/fastq/ERR159/025/ERR15948925/ERR15948925_1.fastq.gz;ftp.sra.ebi.ac.uk/vol1/fastq/ERR159/025/ERR15948925/ERR15948925_2.fastq.gz
171	G18003373	ERR15948927	SAMEA120532923	1313	ftp.sra.ebi.ac.uk/vol1/fastq/ERR159/027/ERR15948927/ERR15948927_1.fastq.gz;ftp.sra.ebi.ac.uk/vol1/fastq/ERR159/027/ERR15948927/ERR15948927_2.fastq.gz
172	G18003372	ERR15948928	SAMEA120532922	1313	ftp.sra.ebi.ac.uk/vol1/fastq/ERR159/028/ERR15948928/ERR15948928_1.fastq.gz;ftp.sra.ebi.ac.uk/vol1/fastq/ERR159/028/ERR15948928/ERR15948928_2.fastq.gz
173	G18003362	ERR15948932	SAMEA120532918	1313	ftp.sra.ebi.ac.uk/vol1/fastq/ERR159/032/ERR15948932/ERR15948932_1.fastq.gz;ftp.sra.ebi.ac.uk/vol1/fastq/ERR159/032/ERR15948932/ERR15948932_2.fastq.gz
174	G18003358	ERR15948933	SAMEA120532917	1313	ftp.sra.ebi.ac.uk/vol1/fastq/ERR159/033/ERR15948933/ERR15948933_1.fastq.gz;ftp.sra.ebi.ac.uk/vol1/fastq/ERR159/033/ERR15948933/ERR15948933_2.fastq.gz
175	G18003317	ERR15948946	SAMEA120532904	1313	ftp.sra.ebi.ac.uk/vol1/fastq/ERR159/046/ERR15948946/ERR15948946_1.fastq.gz;ftp.sra.ebi.ac.uk/vol1/fastq/ERR159/046/ERR15948946/ERR15948946_2.fastq.gz
176	G18003315	ERR15948948	SAMEA120532902	1313	ftp.sra.ebi.ac.uk/vol1/fastq/ERR159/048/ERR15948948/ERR15948948_1.fastq.gz;ftp.sra.ebi.ac.uk/vol1/fastq/ERR159/048/ERR15948948/ERR15948948_2.fastq.gz
177	G18003313	ERR15948949	SAMEA120532901	1313	ftp.sra.ebi.ac.uk/vol1/fastq/ERR159/049/ERR15948949/ERR15948949_1.fastq.gz;ftp.sra.ebi.ac.uk/vol1/fastq/ERR159/049/ERR15948949/ERR15948949_2.fastq.gz
178	G18003312	ERR15948950	SAMEA120532900	1313	ftp.sra.ebi.ac.uk/vol1/fastq/ERR159/050/ERR15948950/ERR15948950_1.fastq.gz;ftp.sra.ebi.ac.uk/vol1/fastq/ERR159/050/ERR15948950/ERR15948950_2.fastq.gz
179	G18003308	ERR15948951	SAMEA120532899	1313	ftp.sra.ebi.ac.uk/vol1/fastq/ERR159/051/ERR15948951/ERR15948951_1.fastq.gz;ftp.sra.ebi.ac.uk/vol1/fastq/ERR159/051/ERR15948951/ERR15948951_2.fastq.gz
180	G18003287	ERR15948955	SAMEA120532895	1313	ftp.sra.ebi.ac.uk/vol1/fastq/ERR159/055/ERR15948955/ERR15948955_1.fastq.gz;ftp.sra.ebi.ac.uk/vol1/fastq/ERR159/055/ERR15948955/ERR15948955_2.fastq.gz
181	G18003285	ERR15948956	SAMEA120532894	1313	ftp.sra.ebi.ac.uk/vol1/fastq/ERR159/056/ERR15948956/ERR15948956_1.fastq.gz;ftp.sra.ebi.ac.uk/vol1/fastq/ERR159/056/ERR15948956/ERR15948956_2.fastq.gz
182	G18003282	ERR15948958	SAMEA120532892	1313	ftp.sra.ebi.ac.uk/vol1/fastq/ERR159/058/ERR15948958/ERR15948958_1.fastq.gz;ftp.sra.ebi.ac.uk/vol1/fastq/ERR159/058/ERR15948958/ERR15948958_2.fastq.gz
183	G18003281	ERR15948959	SAMEA120532891	1313	ftp.sra.ebi.ac.uk/vol1/fastq/ERR159/059/ERR15948959/ERR15948959_1.fastq.gz;ftp.sra.ebi.ac.uk/vol1/fastq/ERR159/059/ERR15948959/ERR15948959_2.fastq.gz
184	G18003257	ERR15948967	SAMEA120532883	1313	ftp.sra.ebi.ac.uk/vol1/fastq/ERR159/067/ERR15948967/ERR15948967_1.fastq.gz;ftp.sra.ebi.ac.uk/vol1/fastq/ERR159/067/ERR15948967/ERR15948967_2.fastq.gz
185	G18003252	ERR15948970	SAMEA120532880	1313	ftp.sra.ebi.ac.uk/vol1/fastq/ERR159/070/ERR15948970/ERR15948970_1.fastq.gz;ftp.sra.ebi.ac.uk/vol1/fastq/ERR159/070/ERR15948970/ERR15948970_2.fastq.gz
186	G18003249	ERR15948973	SAMEA120532877	1313	ftp.sra.ebi.ac.uk/vol1/fastq/ERR159/073/ERR15948973/ERR15948973_1.fastq.gz;ftp.sra.ebi.ac.uk/vol1/fastq/ERR159/073/ERR15948973/ERR15948973_2.fastq.gz
187	G18003521	ERR15948982	SAMEA120532972	1313	ftp.sra.ebi.ac.uk/vol1/fastq/ERR159/082/ERR15948982/ERR15948982_1.fastq.gz;ftp.sra.ebi.ac.uk/vol1/fastq/ERR159/082/ERR15948982/ERR15948982_2.fastq.gz
188	G18003514	ERR15948988	SAMEA120532966	1313	ftp.sra.ebi.ac.uk/vol1/fastq/ERR159/088/ERR15948988/ERR15948988_1.fastq.gz;ftp.sra.ebi.ac.uk/vol1/fastq/ERR159/088/ERR15948988/ERR15948988_2.fastq.gz

189	G20000373	ERR15948697	SAMEA120533180	1313	ftp.sra.ebi.ac.uk/vol1/fastq/ERR159/097/ERR15948697/ERR15948697_1.fastq.gz;ftp.sra.ebi.ac.uk/vol1/fastq/ERR159/097/ERR15948697/ERR15948697_2.fastq.gz
190	G20000346	ERR15948711	SAMEA120533166	1313	ftp.sra.ebi.ac.uk/vol1/fastq/ERR159/011/ERR15948711/ERR15948711_1.fastq.gz;ftp.sra.ebi.ac.uk/vol1/fastq/ERR159/011/ERR15948711/ERR15948711_2.fastq.gz
191	G20000323	ERR15948726	SAMEA120533151	1313	ftp.sra.ebi.ac.uk/vol1/fastq/ERR159/026/ERR15948726/ERR15948726_1.fastq.gz;ftp.sra.ebi.ac.uk/vol1/fastq/ERR159/026/ERR15948726/ERR15948726_2.fastq.gz
192	G20000313	ERR15948732	SAMEA120533145	1313	ftp.sra.ebi.ac.uk/vol1/fastq/ERR159/032/ERR15948732/ERR15948732_1.fastq.gz;ftp.sra.ebi.ac.uk/vol1/fastq/ERR159/032/ERR15948732/ERR15948732_2.fastq.gz
193	G20000285	ERR15948749	SAMEA120533128	1313	ftp.sra.ebi.ac.uk/vol1/fastq/ERR159/049/ERR15948749/ERR15948749_1.fastq.gz;ftp.sra.ebi.ac.uk/vol1/fastq/ERR159/049/ERR15948749/ERR15948749_2.fastq.gz
194	G20000273	ERR15948757	SAMEA120533120	1313	ftp.sra.ebi.ac.uk/vol1/fastq/ERR159/057/ERR15948757/ERR15948757_1.fastq.gz;ftp.sra.ebi.ac.uk/vol1/fastq/ERR159/057/ERR15948757/ERR15948757_2.fastq.gz
195	G20000242	ERR15948772	SAMEA120533105	1313	ftp.sra.ebi.ac.uk/vol1/fastq/ERR159/072/ERR15948772/ERR15948772_1.fastq.gz;ftp.sra.ebi.ac.uk/vol1/fastq/ERR159/072/ERR15948772/ERR15948772_2.fastq.gz
196	G20000219	ERR15948786	SAMEA120533091	1313	ftp.sra.ebi.ac.uk/vol1/fastq/ERR159/086/ERR15948786/ERR15948786_1.fastq.gz;ftp.sra.ebi.ac.uk/vol1/fastq/ERR159/086/ERR15948786/ERR15948786_2.fastq.gz
197	G18003512	ERR15948989	SAMEA120532965	1313	ftp.sra.ebi.ac.uk/vol1/fastq/ERR159/089/ERR15948989/ERR15948989_1.fastq.gz;ftp.sra.ebi.ac.uk/vol1/fastq/ERR159/089/ERR15948989/ERR15948989_2.fastq.gz
198	G18003503	ERR15948991	SAMEA120532963	1313	ftp.sra.ebi.ac.uk/vol1/fastq/ERR159/091/ERR15948991/ERR15948991_1.fastq.gz;ftp.sra.ebi.ac.uk/vol1/fastq/ERR159/091/ERR15948991/ERR15948991_2.fastq.gz
199	G18004011	ERR15948803	SAMEA120533074	1313	ftp.sra.ebi.ac.uk/vol1/fastq/ERR159/003/ERR15948803/ERR15948803_1.fastq.gz;ftp.sra.ebi.ac.uk/vol1/fastq/ERR159/003/ERR15948803/ERR15948803_2.fastq.gz
200	G18003486	ERR15948995	SAMEA120532959	1313	ftp.sra.ebi.ac.uk/vol1/fastq/ERR159/095/ERR15948995/ERR15948995_1.fastq.gz;ftp.sra.ebi.ac.uk/vol1/fastq/ERR159/095/ERR15948995/ERR15948995_2.fastq.gz
201	G18004002	ERR15948806	SAMEA120533071	1313	ftp.sra.ebi.ac.uk/vol1/fastq/ERR159/006/ERR15948806/ERR15948806_1.fastq.gz;ftp.sra.ebi.ac.uk/vol1/fastq/ERR159/006/ERR15948806/ERR15948806_2.fastq.gz
202	G18003467	ERR15949000	SAMEA120532954	1313	ftp.sra.ebi.ac.uk/vol1/fastq/ERR159/000/ERR15949000/ERR15949000_1.fastq.gz;ftp.sra.ebi.ac.uk/vol1/fastq/ERR159/000/ERR15949000/ERR15949000_2.fastq.gz
203	G18003970	ERR15948810	SAMEA120533067	1313	ftp.sra.ebi.ac.uk/vol1/fastq/ERR159/010/ERR15948810/ERR15948810_1.fastq.gz;ftp.sra.ebi.ac.uk/vol1/fastq/ERR159/010/ERR15948810/ERR15948810_2.fastq.gz
204	G18003968	ERR15948811	SAMEA120533066	1313	ftp.sra.ebi.ac.uk/vol1/fastq/ERR159/011/ERR15948811/ERR15948811_1.fastq.gz;ftp.sra.ebi.ac.uk/vol1/fastq/ERR159/011/ERR15948811/ERR15948811_2.fastq.gz
205	G18003463	ERR15949002	SAMEA120532952	1313	ftp.sra.ebi.ac.uk/vol1/fastq/ERR159/002/ERR15949002/ERR15949002_1.fastq.gz;ftp.sra.ebi.ac.uk/vol1/fastq/ERR159/002/ERR15949002/ERR15949002_2.fastq.gz
206	G18003955	ERR15948813	SAMEA120533064	1313	ftp.sra.ebi.ac.uk/vol1/fastq/ERR159/013/ERR15948813/ERR15948813_1.fastq.gz;ftp.sra.ebi.ac.uk/vol1/fastq/ERR159/013/ERR15948813/ERR15948813_2.fastq.gz
207	G18003949	ERR15948814	SAMEA120533063	1313	ftp.sra.ebi.ac.uk/vol1/fastq/ERR159/014/ERR15948814/ERR15948814_1.fastq.gz;ftp.sra.ebi.ac.uk/vol1/fastq/ERR159/014/ERR15948814/ERR15948814_2.fastq.gz
208	G20000383	ERR15948693	SAMEA120533184	1313	ftp.sra.ebi.ac.uk/vol1/fastq/ERR159/093/ERR15948693/ERR15948693_1.fastq.gz;ftp.sra.ebi.ac.uk/vol1/fastq/ERR159/093/ERR15948693/ERR15948693_2.fastq.gz
209	G18003939	ERR15948816	SAMEA120533061	1313	ftp.sra.ebi.ac.uk/vol1/fastq/ERR159/016/ERR15948816/ERR15948816_1.fastq.gz;ftp.sra.ebi.ac.uk/vol1/fastq/ERR159/016/ERR15948816/ERR15948816_2.fastq.gz

210	G18003912	ERR15948824	SAMEA120533053	1313	ftp.sra.ebi.ac.uk/vol1/fastq/ERR159/024/ERR15948824/ERR15948824_1.fastq.gz;ftp.sra.ebi.ac.uk/vol1/fastq/ERR159/024/ERR15948824/ERR15948824_2.fastq.gz
211	G20000366	ERR15948701	SAMEA120533176	1313	ftp.sra.ebi.ac.uk/vol1/fastq/ERR159/001/ERR15948701/ERR15948701_1.fastq.gz;ftp.sra.ebi.ac.uk/vol1/fastq/ERR159/001/ERR15948701/ERR15948701_2.fastq.gz
212	G18003908	ERR15948826	SAMEA120533051	1313	ftp.sra.ebi.ac.uk/vol1/fastq/ERR159/026/ERR15948826/ERR15948826_1.fastq.gz;ftp.sra.ebi.ac.uk/vol1/fastq/ERR159/026/ERR15948826/ERR15948826_2.fastq.gz
213	G20000360	ERR15948704	SAMEA120533173	1313	ftp.sra.ebi.ac.uk/vol1/fastq/ERR159/004/ERR15948704/ERR15948704_1.fastq.gz;ftp.sra.ebi.ac.uk/vol1/fastq/ERR159/004/ERR15948704/ERR15948704_2.fastq.gz
214	G20000336	ERR15948718	SAMEA120533159	1313	ftp.sra.ebi.ac.uk/vol1/fastq/ERR159/018/ERR15948718/ERR15948718_1.fastq.gz;ftp.sra.ebi.ac.uk/vol1/fastq/ERR159/018/ERR15948718/ERR15948718_2.fastq.gz
215	G18003906	ERR15948827	SAMEA120533050	1313	ftp.sra.ebi.ac.uk/vol1/fastq/ERR159/027/ERR15948827/ERR15948827_1.fastq.gz;ftp.sra.ebi.ac.uk/vol1/fastq/ERR159/027/ERR15948827/ERR15948827_2.fastq.gz
216	G18003905	ERR15948828	SAMEA120533049	1313	ftp.sra.ebi.ac.uk/vol1/fastq/ERR159/028/ERR15948828/ERR15948828_1.fastq.gz;ftp.sra.ebi.ac.uk/vol1/fastq/ERR159/028/ERR15948828/ERR15948828_2.fastq.gz
217	G20000334	ERR15948720	SAMEA120533157	1313	ftp.sra.ebi.ac.uk/vol1/fastq/ERR159/020/ERR15948720/ERR15948720_1.fastq.gz;ftp.sra.ebi.ac.uk/vol1/fastq/ERR159/020/ERR15948720/ERR15948720_2.fastq.gz
218	G18003894	ERR15948831	SAMEA120533046	1313	ftp.sra.ebi.ac.uk/vol1/fastq/ERR159/031/ERR15948831/ERR15948831_1.fastq.gz;ftp.sra.ebi.ac.uk/vol1/fastq/ERR159/031/ERR15948831/ERR15948831_2.fastq.gz
219	G18003885	ERR15948834	SAMEA120533043	1313	ftp.sra.ebi.ac.uk/vol1/fastq/ERR159/034/ERR15948834/ERR15948834_1.fastq.gz;ftp.sra.ebi.ac.uk/vol1/fastq/ERR159/034/ERR15948834/ERR15948834_2.fastq.gz
220	G18003853	ERR15948841	SAMEA120533036	1313	ftp.sra.ebi.ac.uk/vol1/fastq/ERR159/041/ERR15948841/ERR15948841_1.fastq.gz;ftp.sra.ebi.ac.uk/vol1/fastq/ERR159/041/ERR15948841/ERR15948841_2.fastq.gz
221	G20000330	ERR15948721	SAMEA120533156	1313	ftp.sra.ebi.ac.uk/vol1/fastq/ERR159/021/ERR15948721/ERR15948721_1.fastq.gz;ftp.sra.ebi.ac.uk/vol1/fastq/ERR159/021/ERR15948721/ERR15948721_2.fastq.gz
222	G18003831	ERR15948843	SAMEA120533034	1313	ftp.sra.ebi.ac.uk/vol1/fastq/ERR159/043/ERR15948843/ERR15948843_1.fastq.gz;ftp.sra.ebi.ac.uk/vol1/fastq/ERR159/043/ERR15948843/ERR15948843_2.fastq.gz
223	G18003803	ERR15948853	SAMEA120533024	1313	ftp.sra.ebi.ac.uk/vol1/fastq/ERR159/053/ERR15948853/ERR15948853_1.fastq.gz;ftp.sra.ebi.ac.uk/vol1/fastq/ERR159/053/ERR15948853/ERR15948853_2.fastq.gz
224	G20000328	ERR15948723	SAMEA120533154	1313	ftp.sra.ebi.ac.uk/vol1/fastq/ERR159/023/ERR15948723/ERR15948723_1.fastq.gz;ftp.sra.ebi.ac.uk/vol1/fastq/ERR159/023/ERR15948723/ERR15948723_2.fastq.gz
225	G18003792	ERR15948859	SAMEA120533018	1313	ftp.sra.ebi.ac.uk/vol1/fastq/ERR159/059/ERR15948859/ERR15948859_1.fastq.gz;ftp.sra.ebi.ac.uk/vol1/fastq/ERR159/059/ERR15948859/ERR15948859_2.fastq.gz
226	G18003770	ERR15948863	SAMEA120533014	1313	ftp.sra.ebi.ac.uk/vol1/fastq/ERR159/063/ERR15948863/ERR15948863_1.fastq.gz;ftp.sra.ebi.ac.uk/vol1/fastq/ERR159/063/ERR15948863/ERR15948863_2.fastq.gz
227	G20000327	ERR15948724	SAMEA120533153	1313	ftp.sra.ebi.ac.uk/vol1/fastq/ERR159/024/ERR15948724/ERR15948724_1.fastq.gz;ftp.sra.ebi.ac.uk/vol1/fastq/ERR159/024/ERR15948724/ERR15948724_2.fastq.gz
228	G18003692	ERR15948871	SAMEA120533006	1313	ftp.sra.ebi.ac.uk/vol1/fastq/ERR159/071/ERR15948871/ERR15948871_1.fastq.gz;ftp.sra.ebi.ac.uk/vol1/fastq/ERR159/071/ERR15948871/ERR15948871_2.fastq.gz
229	G18003688	ERR15948872	SAMEA120533005	1313	ftp.sra.ebi.ac.uk/vol1/fastq/ERR159/072/ERR15948872/ERR15948872_1.fastq.gz;ftp.sra.ebi.ac.uk/vol1/fastq/ERR159/072/ERR15948872/ERR15948872_2.fastq.gz
230	G20000322	ERR15948727	SAMEA120533150	1313	ftp.sra.ebi.ac.uk/vol1/fastq/ERR159/027/ERR15948727/ERR15948727_1.fastq.gz;ftp.sra.ebi.ac.uk/vol1/fastq/ERR159/027/ERR15948727/ERR15948727_2.fastq.gz

231	G18003684	ERR15948873	SAMEA120533004	1313	ftp.sra.ebi.ac.uk/vol1/fastq/ERR159/073/ERR15948873/ERR15948873_1.fastq.gz;ftp.sra.ebi.ac.uk/vol1/fastq/ERR159/073/ERR15948873/ERR15948873_2.fastq.gz
232	G18003674	ERR15948877	SAMEA120533000	1313	ftp.sra.ebi.ac.uk/vol1/fastq/ERR159/077/ERR15948877/ERR15948877_1.fastq.gz;ftp.sra.ebi.ac.uk/vol1/fastq/ERR159/077/ERR15948877/ERR15948877_2.fastq.gz
233	G20000317	ERR15948730	SAMEA120533147	1313	ftp.sra.ebi.ac.uk/vol1/fastq/ERR159/030/ERR15948730/ERR15948730_1.fastq.gz;ftp.sra.ebi.ac.uk/vol1/fastq/ERR159/030/ERR15948730/ERR15948730_2.fastq.gz
234	G18003659	ERR15948880	SAMEA120532997	1313	ftp.sra.ebi.ac.uk/vol1/fastq/ERR159/080/ERR15948880/ERR15948880_1.fastq.gz;ftp.sra.ebi.ac.uk/vol1/fastq/ERR159/080/ERR15948880/ERR15948880_2.fastq.gz
235	G20000307	ERR15948736	SAMEA120533141	1313	ftp.sra.ebi.ac.uk/vol1/fastq/ERR159/036/ERR15948736/ERR15948736_1.fastq.gz;ftp.sra.ebi.ac.uk/vol1/fastq/ERR159/036/ERR15948736/ERR15948736_2.fastq.gz
236	G18003600	ERR15948882	SAMEA120532995	1313	ftp.sra.ebi.ac.uk/vol1/fastq/ERR159/082/ERR15948882/ERR15948882_1.fastq.gz;ftp.sra.ebi.ac.uk/vol1/fastq/ERR159/082/ERR15948882/ERR15948882_2.fastq.gz
237	G20000295	ERR15948741	SAMEA120533136	1313	ftp.sra.ebi.ac.uk/vol1/fastq/ERR159/041/ERR15948741/ERR15948741_1.fastq.gz;ftp.sra.ebi.ac.uk/vol1/fastq/ERR159/041/ERR15948741/ERR15948741_2.fastq.gz
238	G20000288	ERR15948748	SAMEA120533129	1313	ftp.sra.ebi.ac.uk/vol1/fastq/ERR159/048/ERR15948748/ERR15948748_1.fastq.gz;ftp.sra.ebi.ac.uk/vol1/fastq/ERR159/048/ERR15948748/ERR15948748_2.fastq.gz
239	G20000263	ERR15948763	SAMEA120533114	1313	ftp.sra.ebi.ac.uk/vol1/fastq/ERR159/063/ERR15948763/ERR15948763_1.fastq.gz;ftp.sra.ebi.ac.uk/vol1/fastq/ERR159/063/ERR15948763/ERR15948763_2.fastq.gz
240	G18003567	ERR15948890	SAMEA120532987	1313	ftp.sra.ebi.ac.uk/vol1/fastq/ERR159/090/ERR15948890/ERR15948890_1.fastq.gz;ftp.sra.ebi.ac.uk/vol1/fastq/ERR159/090/ERR15948890/ERR15948890_2.fastq.gz
241	G18003557	ERR15948894	SAMEA120532983	1313	ftp.sra.ebi.ac.uk/vol1/fastq/ERR159/094/ERR15948894/ERR15948894_1.fastq.gz;ftp.sra.ebi.ac.uk/vol1/fastq/ERR159/094/ERR15948894/ERR15948894_2.fastq.gz
242	G20000233	ERR15948777	SAMEA120533100	1313	ftp.sra.ebi.ac.uk/vol1/fastq/ERR159/077/ERR15948777/ERR15948777_1.fastq.gz;ftp.sra.ebi.ac.uk/vol1/fastq/ERR159/077/ERR15948777/ERR15948777_2.fastq.gz
243	G18003540	ERR15948898	SAMEA120532979	1313	ftp.sra.ebi.ac.uk/vol1/fastq/ERR159/098/ERR15948898/ERR15948898_1.fastq.gz;ftp.sra.ebi.ac.uk/vol1/fastq/ERR159/098/ERR15948898/ERR15948898_2.fastq.gz
244	G20000229	ERR15948780	SAMEA120533097	1313	ftp.sra.ebi.ac.uk/vol1/fastq/ERR159/080/ERR15948780/ERR15948780_1.fastq.gz;ftp.sra.ebi.ac.uk/vol1/fastq/ERR159/080/ERR15948780/ERR15948780_2.fastq.gz
245	G18003535	ERR15948900	SAMEA120532977	1313	ftp.sra.ebi.ac.uk/vol1/fastq/ERR159/000/ERR15948900/ERR15948900_1.fastq.gz;ftp.sra.ebi.ac.uk/vol1/fastq/ERR159/000/ERR15948900/ERR15948900_2.fastq.gz
246	G18003430	ERR15948907	SAMEA120532943	1313	ftp.sra.ebi.ac.uk/vol1/fastq/ERR159/007/ERR15948907/ERR15948907_1.fastq.gz;ftp.sra.ebi.ac.uk/vol1/fastq/ERR159/007/ERR15948907/ERR15948907_2.fastq.gz
247	G20000222	ERR15948783	SAMEA120533094	1313	ftp.sra.ebi.ac.uk/vol1/fastq/ERR159/083/ERR15948783/ERR15948783_1.fastq.gz;ftp.sra.ebi.ac.uk/vol1/fastq/ERR159/083/ERR15948783/ERR15948783_2.fastq.gz
248	G20000207	ERR15948791	SAMEA120533086	1313	ftp.sra.ebi.ac.uk/vol1/fastq/ERR159/091/ERR15948791/ERR15948791_1.fastq.gz;ftp.sra.ebi.ac.uk/vol1/fastq/ERR159/091/ERR15948791/ERR15948791_2.fastq.gz
249	G18003414	ERR15948914	SAMEA120532936	1313	ftp.sra.ebi.ac.uk/vol1/fastq/ERR159/014/ERR15948914/ERR15948914_1.fastq.gz;ftp.sra.ebi.ac.uk/vol1/fastq/ERR159/014/ERR15948914/ERR15948914_2.fastq.gz
250	G18003396	ERR15948917	SAMEA120532933	1313	ftp.sra.ebi.ac.uk/vol1/fastq/ERR159/017/ERR15948917/ERR15948917_1.fastq.gz;ftp.sra.ebi.ac.uk/vol1/fastq/ERR159/017/ERR15948917/ERR15948917_2.fastq.gz
251	G20000203	ERR15948794	SAMEA120533083	1313	ftp.sra.ebi.ac.uk/vol1/fastq/ERR159/094/ERR15948794/ERR15948794_1.fastq.gz;ftp.sra.ebi.ac.uk/vol1/fastq/ERR159/094/ERR15948794/ERR15948794_2.fastq.gz

252	G18003389	ERR15948919	SAMEA120532931	1313	ftp.sra.ebi.ac.uk/vol1/fastq/ERR159/019/ERR15948919/ERR15948919_1.fastq.gz;ftp.sra.ebi.ac.uk/vol1/fastq/ERR159/019/ERR15948919/ERR15948919_2.fastq.gz
253	G18004025	ERR15948797	SAMEA120533080	1313	ftp.sra.ebi.ac.uk/vol1/fastq/ERR159/097/ERR15948797/ERR15948797_1.fastq.gz;ftp.sra.ebi.ac.uk/vol1/fastq/ERR159/097/ERR15948797/ERR15948797_2.fastq.gz
254	G18003383	ERR15948922	SAMEA120532928	1313	ftp.sra.ebi.ac.uk/vol1/fastq/ERR159/022/ERR15948922/ERR15948922_1.fastq.gz;ftp.sra.ebi.ac.uk/vol1/fastq/ERR159/022/ERR15948922/ERR15948922_2.fastq.gz
255	G18004016	ERR15948801	SAMEA120533076	1313	ftp.sra.ebi.ac.uk/vol1/fastq/ERR159/001/ERR15948801/ERR15948801_1.fastq.gz;ftp.sra.ebi.ac.uk/vol1/fastq/ERR159/001/ERR15948801/ERR15948801_2.fastq.gz
256	G18003378	ERR15948923	SAMEA120532927	1313	ftp.sra.ebi.ac.uk/vol1/fastq/ERR159/023/ERR15948923/ERR15948923_1.fastq.gz;ftp.sra.ebi.ac.uk/vol1/fastq/ERR159/023/ERR15948923/ERR15948923_2.fastq.gz
257	G18003377	ERR15948924	SAMEA120532926	1313	ftp.sra.ebi.ac.uk/vol1/fastq/ERR159/024/ERR15948924/ERR15948924_1.fastq.gz;ftp.sra.ebi.ac.uk/vol1/fastq/ERR159/024/ERR15948924/ERR15948924_2.fastq.gz
258	G20000335	ERR15948719	SAMEA120533158	1313	ftp.sra.ebi.ac.uk/vol1/fastq/ERR159/019/ERR15948719/ERR15948719_1.fastq.gz;ftp.sra.ebi.ac.uk/vol1/fastq/ERR159/019/ERR15948719/ERR15948719_2.fastq.gz
259	G18003375	ERR15948926	SAMEA120532924	1313	ftp.sra.ebi.ac.uk/vol1/fastq/ERR159/026/ERR15948926/ERR15948926_1.fastq.gz;ftp.sra.ebi.ac.uk/vol1/fastq/ERR159/026/ERR15948926/ERR15948926_2.fastq.gz
260	G18003349	ERR15948935	SAMEA120532915	1313	ftp.sra.ebi.ac.uk/vol1/fastq/ERR159/035/ERR15948935/ERR15948935_1.fastq.gz;ftp.sra.ebi.ac.uk/vol1/fastq/ERR159/035/ERR15948935/ERR15948935_2.fastq.gz
261	G18003335	ERR15948937	SAMEA120532913	1313	ftp.sra.ebi.ac.uk/vol1/fastq/ERR159/037/ERR15948937/ERR15948937_1.fastq.gz;ftp.sra.ebi.ac.uk/vol1/fastq/ERR159/037/ERR15948937/ERR15948937_2.fastq.gz
262	G18003332	ERR15948938	SAMEA120532912	1313	ftp.sra.ebi.ac.uk/vol1/fastq/ERR159/038/ERR15948938/ERR15948938_1.fastq.gz;ftp.sra.ebi.ac.uk/vol1/fastq/ERR159/038/ERR15948938/ERR15948938_2.fastq.gz
263	G18003331	ERR15948939	SAMEA120532911	1313	ftp.sra.ebi.ac.uk/vol1/fastq/ERR159/039/ERR15948939/ERR15948939_1.fastq.gz;ftp.sra.ebi.ac.uk/vol1/fastq/ERR159/039/ERR15948939/ERR15948939_2.fastq.gz
264	G18003327	ERR15948940	SAMEA120532910	1313	ftp.sra.ebi.ac.uk/vol1/fastq/ERR159/040/ERR15948940/ERR15948940_1.fastq.gz;ftp.sra.ebi.ac.uk/vol1/fastq/ERR159/040/ERR15948940/ERR15948940_2.fastq.gz
265	G18003323	ERR15948942	SAMEA120532908	1313	ftp.sra.ebi.ac.uk/vol1/fastq/ERR159/042/ERR15948942/ERR15948942_1.fastq.gz;ftp.sra.ebi.ac.uk/vol1/fastq/ERR159/042/ERR15948942/ERR15948942_2.fastq.gz
266	G18003297	ERR15948953	SAMEA120532897	1313	ftp.sra.ebi.ac.uk/vol1/fastq/ERR159/053/ERR15948953/ERR15948953_1.fastq.gz;ftp.sra.ebi.ac.uk/vol1/fastq/ERR159/053/ERR15948953/ERR15948953_2.fastq.gz
267	G18003279	ERR15948960	SAMEA120532890	1313	ftp.sra.ebi.ac.uk/vol1/fastq/ERR159/060/ERR15948960/ERR15948960_1.fastq.gz;ftp.sra.ebi.ac.uk/vol1/fastq/ERR159/060/ERR15948960/ERR15948960_2.fastq.gz
268	G18003275	ERR15948961	SAMEA120532889	1313	ftp.sra.ebi.ac.uk/vol1/fastq/ERR159/061/ERR15948961/ERR15948961_1.fastq.gz;ftp.sra.ebi.ac.uk/vol1/fastq/ERR159/061/ERR15948961/ERR15948961_2.fastq.gz
269	G18003269	ERR15948963	SAMEA120532887	1313	ftp.sra.ebi.ac.uk/vol1/fastq/ERR159/063/ERR15948963/ERR15948963_1.fastq.gz;ftp.sra.ebi.ac.uk/vol1/fastq/ERR159/063/ERR15948963/ERR15948963_2.fastq.gz
270	G18003258	ERR15948966	SAMEA120532884	1313	ftp.sra.ebi.ac.uk/vol1/fastq/ERR159/066/ERR15948966/ERR15948966_1.fastq.gz;ftp.sra.ebi.ac.uk/vol1/fastq/ERR159/066/ERR15948966/ERR15948966_2.fastq.gz
271	G18003255	ERR15948968	SAMEA120532882	1313	ftp.sra.ebi.ac.uk/vol1/fastq/ERR159/068/ERR15948968/ERR15948968_1.fastq.gz;ftp.sra.ebi.ac.uk/vol1/fastq/ERR159/068/ERR15948968/ERR15948968_2.fastq.gz
272	G18003254	ERR15948969	SAMEA120532881	1313	ftp.sra.ebi.ac.uk/vol1/fastq/ERR159/069/ERR15948969/ERR15948969_1.fastq.gz;ftp.sra.ebi.ac.uk/vol1/fastq/ERR159/069/ERR15948969/ERR15948969_2.fastq.gz

273	G18003247	ERR15948975	SAMEA120532875	1313	ftp.sra.ebi.ac.uk/vol1/fastq/ERR159/075/ERR15948975/ERR15948975_1.fastq.gz;ftp.sra.ebi.ac.uk/vol1/fastq/ERR159/075/ERR15948975/ERR15948975_2.fastq.gz
274	G18003246	ERR15948976	SAMEA120532874	1313	ftp.sra.ebi.ac.uk/vol1/fastq/ERR159/076/ERR15948976/ERR15948976_1.fastq.gz;ftp.sra.ebi.ac.uk/vol1/fastq/ERR159/076/ERR15948976/ERR15948976_2.fastq.gz
275	G18003530	ERR15948979	SAMEA120532975	1313	ftp.sra.ebi.ac.uk/vol1/fastq/ERR159/079/ERR15948979/ERR15948979_1.fastq.gz;ftp.sra.ebi.ac.uk/vol1/fastq/ERR159/079/ERR15948979/ERR15948979_2.fastq.gz
276	G18003528	ERR15948980	SAMEA120532974	1313	ftp.sra.ebi.ac.uk/vol1/fastq/ERR159/080/ERR15948980/ERR15948980_1.fastq.gz;ftp.sra.ebi.ac.uk/vol1/fastq/ERR159/080/ERR15948980/ERR15948980_2.fastq.gz
277	G18003523	ERR15948981	SAMEA120532973	1313	ftp.sra.ebi.ac.uk/vol1/fastq/ERR159/081/ERR15948981/ERR15948981_1.fastq.gz;ftp.sra.ebi.ac.uk/vol1/fastq/ERR159/081/ERR15948981/ERR15948981_2.fastq.gz
278	G18003520	ERR15948983	SAMEA120532971	1313	ftp.sra.ebi.ac.uk/vol1/fastq/ERR159/083/ERR15948983/ERR15948983_1.fastq.gz;ftp.sra.ebi.ac.uk/vol1/fastq/ERR159/083/ERR15948983/ERR15948983_2.fastq.gz
279	G18003519	ERR15948984	SAMEA120532970	1313	ftp.sra.ebi.ac.uk/vol1/fastq/ERR159/084/ERR15948984/ERR15948984_1.fastq.gz;ftp.sra.ebi.ac.uk/vol1/fastq/ERR159/084/ERR15948984/ERR15948984_2.fastq.gz
280	G18003516	ERR15948987	SAMEA120532967	1313	ftp.sra.ebi.ac.uk/vol1/fastq/ERR159/087/ERR15948987/ERR15948987_1.fastq.gz;ftp.sra.ebi.ac.uk/vol1/fastq/ERR159/087/ERR15948987/ERR15948987_2.fastq.gz
281	G18003506	ERR15948990	SAMEA120532964	1313	ftp.sra.ebi.ac.uk/vol1/fastq/ERR159/090/ERR15948990/ERR15948990_1.fastq.gz;ftp.sra.ebi.ac.uk/vol1/fastq/ERR159/090/ERR15948990/ERR15948990_2.fastq.gz
282	G18003485	ERR15948996	SAMEA120532958	1313	ftp.sra.ebi.ac.uk/vol1/fastq/ERR159/096/ERR15948996/ERR15948996_1.fastq.gz;ftp.sra.ebi.ac.uk/vol1/fastq/ERR159/096/ERR15948996/ERR15948996_2.fastq.gz
283	G18003484	ERR15948997	SAMEA120532957	1313	ftp.sra.ebi.ac.uk/vol1/fastq/ERR159/097/ERR15948997/ERR15948997_1.fastq.gz;ftp.sra.ebi.ac.uk/vol1/fastq/ERR159/097/ERR15948997/ERR15948997_2.fastq.gz
284	G18003471	ERR15948999	SAMEA120532955	1313	ftp.sra.ebi.ac.uk/vol1/fastq/ERR159/099/ERR15948999/ERR15948999_1.fastq.gz;ftp.sra.ebi.ac.uk/vol1/fastq/ERR159/099/ERR15948999/ERR15948999_2.fastq.gz
285	G18003466	ERR15949001	SAMEA120532953	1313	ftp.sra.ebi.ac.uk/vol1/fastq/ERR159/001/ERR15949001/ERR15949001_1.fastq.gz;ftp.sra.ebi.ac.uk/vol1/fastq/ERR159/001/ERR15949001/ERR15949001_2.fastq.gz
286	G18003457	ERR15949004	SAMEA120532950	1313	ftp.sra.ebi.ac.uk/vol1/fastq/ERR159/004/ERR15949004/ERR15949004_1.fastq.gz;ftp.sra.ebi.ac.uk/vol1/fastq/ERR159/004/ERR15949004/ERR15949004_2.fastq.gz
287	G20000367	ERR15948700	SAMEA120533177	1313	ftp.sra.ebi.ac.uk/vol1/fastq/ERR159/000/ERR15948700/ERR15948700_1.fastq.gz;ftp.sra.ebi.ac.uk/vol1/fastq/ERR159/000/ERR15948700/ERR15948700_2.fastq.gz
288	G20000356	ERR15948706	SAMEA120533171	1313	ftp.sra.ebi.ac.uk/vol1/fastq/ERR159/006/ERR15948706/ERR15948706_1.fastq.gz;ftp.sra.ebi.ac.uk/vol1/fastq/ERR159/006/ERR15948706/ERR15948706_2.fastq.gz
289	G20000347	ERR15948710	SAMEA120533167	1313	ftp.sra.ebi.ac.uk/vol1/fastq/ERR159/010/ERR15948710/ERR15948710_1.fastq.gz;ftp.sra.ebi.ac.uk/vol1/fastq/ERR159/010/ERR15948710/ERR15948710_2.fastq.gz
290	G20000345	ERR15948712	SAMEA120533165	1313	ftp.sra.ebi.ac.uk/vol1/fastq/ERR159/012/ERR15948712/ERR15948712_1.fastq.gz;ftp.sra.ebi.ac.uk/vol1/fastq/ERR159/012/ERR15948712/ERR15948712_2.fastq.gz
291	G20000342	ERR15948715	SAMEA120533162	1313	ftp.sra.ebi.ac.uk/vol1/fastq/ERR159/015/ERR15948715/ERR15948715_1.fastq.gz;ftp.sra.ebi.ac.uk/vol1/fastq/ERR159/015/ERR15948715/ERR15948715_2.fastq.gz
292	G20000339	ERR15948716	SAMEA120533161	1313	ftp.sra.ebi.ac.uk/vol1/fastq/ERR159/016/ERR15948716/ERR15948716_1.fastq.gz;ftp.sra.ebi.ac.uk/vol1/fastq/ERR159/016/ERR15948716/ERR15948716_2.fastq.gz
293	G20000329	ERR15948722	SAMEA120533155	1313	ftp.sra.ebi.ac.uk/vol1/fastq/ERR159/022/ERR15948722/ERR15948722_1.fastq.gz;ftp.sra.ebi.ac.uk/vol1/fastq/ERR159/022/ERR15948722/ERR15948722_2.fastq.gz

294	G20000312	ERR15948733	SAMEA120533144	1313	ftp.sra.ebi.ac.uk/vol1/fastq/ERR159/033/ERR15948733/ERR15948733_1.fastq.gz;ftp.sra.ebi.ac.uk/vol1/fastq/ERR159/033/ERR15948733/ERR15948733_2.fastq.gz
295	G20000310	ERR15948735	SAMEA120533142	1313	ftp.sra.ebi.ac.uk/vol1/fastq/ERR159/035/ERR15948735/ERR15948735_1.fastq.gz;ftp.sra.ebi.ac.uk/vol1/fastq/ERR159/035/ERR15948735/ERR15948735_2.fastq.gz
296	G20000298	ERR15948739	SAMEA120533138	1313	ftp.sra.ebi.ac.uk/vol1/fastq/ERR159/039/ERR15948739/ERR15948739_1.fastq.gz;ftp.sra.ebi.ac.uk/vol1/fastq/ERR159/039/ERR15948739/ERR15948739_2.fastq.gz
297	G20000294	ERR15948742	SAMEA120533135	1313	ftp.sra.ebi.ac.uk/vol1/fastq/ERR159/042/ERR15948742/ERR15948742_1.fastq.gz;ftp.sra.ebi.ac.uk/vol1/fastq/ERR159/042/ERR15948742/ERR15948742_2.fastq.gz
298	G20000292	ERR15948744	SAMEA120533133	1313	ftp.sra.ebi.ac.uk/vol1/fastq/ERR159/044/ERR15948744/ERR15948744_1.fastq.gz;ftp.sra.ebi.ac.uk/vol1/fastq/ERR159/044/ERR15948744/ERR15948744_2.fastq.gz
299	G20000282	ERR15948750	SAMEA120533127	1313	ftp.sra.ebi.ac.uk/vol1/fastq/ERR159/050/ERR15948750/ERR15948750_1.fastq.gz;ftp.sra.ebi.ac.uk/vol1/fastq/ERR159/050/ERR15948750/ERR15948750_2.fastq.gz
300	G20000281	ERR15948751	SAMEA120533126	1313	ftp.sra.ebi.ac.uk/vol1/fastq/ERR159/051/ERR15948751/ERR15948751_1.fastq.gz;ftp.sra.ebi.ac.uk/vol1/fastq/ERR159/051/ERR15948751/ERR15948751_2.fastq.gz
301	G20000271	ERR15948759	SAMEA120533118	1313	ftp.sra.ebi.ac.uk/vol1/fastq/ERR159/059/ERR15948759/ERR15948759_1.fastq.gz;ftp.sra.ebi.ac.uk/vol1/fastq/ERR159/059/ERR15948759/ERR15948759_2.fastq.gz
302	G20000262	ERR15948764	SAMEA120533113	1313	ftp.sra.ebi.ac.uk/vol1/fastq/ERR159/064/ERR15948764/ERR15948764_1.fastq.gz;ftp.sra.ebi.ac.uk/vol1/fastq/ERR159/064/ERR15948764/ERR15948764_2.fastq.gz
303	G20000257	ERR15948766	SAMEA120533111	1313	ftp.sra.ebi.ac.uk/vol1/fastq/ERR159/066/ERR15948766/ERR15948766_1.fastq.gz;ftp.sra.ebi.ac.uk/vol1/fastq/ERR159/066/ERR15948766/ERR15948766_2.fastq.gz
304	G20000251	ERR15948769	SAMEA120533108	1313	ftp.sra.ebi.ac.uk/vol1/fastq/ERR159/069/ERR15948769/ERR15948769_1.fastq.gz;ftp.sra.ebi.ac.uk/vol1/fastq/ERR159/069/ERR15948769/ERR15948769_2.fastq.gz
305	G20000250	ERR15948770	SAMEA120533107	1313	ftp.sra.ebi.ac.uk/vol1/fastq/ERR159/070/ERR15948770/ERR15948770_1.fastq.gz;ftp.sra.ebi.ac.uk/vol1/fastq/ERR159/070/ERR15948770/ERR15948770_2.fastq.gz
306	G20000247	ERR15948771	SAMEA120533106	1313	ftp.sra.ebi.ac.uk/vol1/fastq/ERR159/071/ERR15948771/ERR15948771_1.fastq.gz;ftp.sra.ebi.ac.uk/vol1/fastq/ERR159/071/ERR15948771/ERR15948771_2.fastq.gz
307	G20000220	ERR15948785	SAMEA120533092	1313	ftp.sra.ebi.ac.uk/vol1/fastq/ERR159/085/ERR15948785/ERR15948785_1.fastq.gz;ftp.sra.ebi.ac.uk/vol1/fastq/ERR159/085/ERR15948785/ERR15948785_2.fastq.gz
308	G20000216	ERR15948787	SAMEA120533090	1313	ftp.sra.ebi.ac.uk/vol1/fastq/ERR159/087/ERR15948787/ERR15948787_1.fastq.gz;ftp.sra.ebi.ac.uk/vol1/fastq/ERR159/087/ERR15948787/ERR15948787_2.fastq.gz
309	G20000215	ERR15948788	SAMEA120533089	1313	ftp.sra.ebi.ac.uk/vol1/fastq/ERR159/088/ERR15948788/ERR15948788_1.fastq.gz;ftp.sra.ebi.ac.uk/vol1/fastq/ERR159/088/ERR15948788/ERR15948788_2.fastq.gz
310	G18004022	ERR15948800	SAMEA120533077	1313	ftp.sra.ebi.ac.uk/vol1/fastq/ERR159/000/ERR15948800/ERR15948800_1.fastq.gz;ftp.sra.ebi.ac.uk/vol1/fastq/ERR159/000/ERR15948800/ERR15948800_2.fastq.gz
311	G18004012	ERR15948802	SAMEA120533075	1313	ftp.sra.ebi.ac.uk/vol1/fastq/ERR159/002/ERR15948802/ERR15948802_1.fastq.gz;ftp.sra.ebi.ac.uk/vol1/fastq/ERR159/002/ERR15948802/ERR15948802_2.fastq.gz
312	G18003721	ERR15948866	SAMEA120533011	1313	ftp.sra.ebi.ac.uk/vol1/fastq/ERR159/066/ERR15948866/ERR15948866_1.fastq.gz;ftp.sra.ebi.ac.uk/vol1/fastq/ERR159/066/ERR15948866/ERR15948866_2.fastq.gz
313	G18003419	ERR15948912	SAMEA120532938	1313	ftp.sra.ebi.ac.uk/vol1/fastq/ERR159/012/ERR15948912/ERR15948912_1.fastq.gz;ftp.sra.ebi.ac.uk/vol1/fastq/ERR159/012/ERR15948912/ERR15948912_2.fastq.gz
